# Metabolic correlates of endoscopy-confirmed reflux esophagitis in hospital-based patients with type 2 diabetes mellitus

**DOI:** 10.3389/fendo.2026.1886576

**Published:** 2026-08-03

**Authors:** Ping Li, Shanshan Wang, Yijie Wang, Xiaojuan Rao, Jingya Dong

**Affiliations:** 1 Department of Endocrinology, the Fifth Affiliated Hospital of Zhengzhou University, Zhengzhou, China; 2 School of Computer and Artificial Intelligence, Zhengzhou University, Zhengzhou, China

**Keywords:** type 2 diabetes mellitus, gastrointestinal polyps, colorectal polyps, adenomatous polyps, fecal occult blood test

## Abstract

**Background:**

Reflux esophagitis (RE) is an endoscopy-defined manifestation of gastroesophageal reflux disease and may be related to metabolic disturbance in type 2 diabetes mellitus (T2DM). The clinical-metabolic characteristics associated with endoscopy-confirmed RE in T2DM remain incompletely defined, particularly with regard to glycemic control, 25-hydroxyvitamin D [25(OH)D] status, and insulin-related indicators. This study evaluates the joint associations of these metabolic factors with endoscopy-confirmed RE in a hospital-based T2DM population.

**Methods:**

The analytic cohort comprises 772 hospital-based patients with T2DM and ascertainable RE status from endoscopic diagnostic records. Demographic characteristics, diabetes-related complications, glycemic indices, lipid parameters, renal function, 25(OH)D, insulin-related indicators, and gastrointestinal test findings are assessed. Prespecified multivariable logistic regression models are applied to identify clinical-metabolic correlates of RE. Restricted cubic spline analyses are used to assess potential nonlinear associations, and exploratory discrimination analyses are performed to evaluate risk-stratification performance.

**Results:**

Among 772 patients, 223 are classified as having RE, corresponding to an observed prevalence of 28.9%. Compared with the non-RE group, the RE group has higher HbA1c, fasting insulin, triglycerides, HOMA-IR, fasting-state ISI, and TyG index, and lower 25(OH)D levels. In fully adjusted models, HbA1c remains consistently associated with RE (adjusted odds ratio [aOR] per 1-standard-deviation increase, 1.58; 95% confidence interval [CI], 1.33-1.87). The inverse association between 25(OH)D and RE attenuates after adjustment for HbA1c and TyG index (aOR, 0.84; 95% CI, 0.70-1.00). The TyG index provides limited additional information after HbA1c adjustment (aOR, 1.02; 95% CI, 0.85-1.21), whereas HOMA-IR, fasting insulin, and fasting-state ISI show positive associations with RE when evaluated separately. Restricted cubic spline analyses indicate nonlinear associations for HbA1c and insulin-related indicators, with stronger metabolic signals at higher value ranges. Exploratory discrimination analyses indicate limited-to-moderate risk-stratification performance.

**Conclusion:**

In hospital-based patients with T2DM, endoscopy-confirmed RE is associated with an adverse clinical-metabolic profile, mainly characterized by higher HbA1c and selected insulin-related abnormalities. The inverse association between 25(OH)D and RE attenuates after glycemic adjustment and requires cautious interpretation. These cross-sectional findings are associational and hypothesis-generating, and prospective studies are needed to clarify temporal relationships and clinical implications.

## 1 Introduction

Type 2 diabetes mellitus (T2DM) is a major chronic metabolic disease with an increasing global burden (1). Beyond hyperglycemia, T2DM is commonly accompanied by obesity, dyslipidemia, hypertension, insulin resistance, and chronic microvascular or macrovascular complications. Clinical management usually focuses on glycemic control and cardiovascular, renal, retinal, and neurological outcomes, whereas upper gastrointestinal manifestations receive less systematic attention. Reflux esophagitis (RE), an endoscopically confirmed phenotype of gastroesophageal reflux disease (GERD), reflects reflux-related mucosal injury and may share metabolic and neurofunctional pathways with diabetes. Clarifying the clinical-metabolic correlates of RE in patients with T2DM is therefore relevant for understanding reflux-associated injury in a metabolically vulnerable population.

Existing studies suggest several links between T2DM, metabolic dysfunction, and reflux-related disease. Diabetic autonomic neuropathy may impair esophageal peristalsis, lower esophageal sphincter function, esophageal clearance, and gastric emptying, thereby increasing reflux exposure and mucosal vulnerability (2, 3) Functional studies using impedance-pH monitoring, esophageal manometry, heart rate variability, and high-resolution manometry further indicate that diabetic patients may have abnormal reflux burden or esophageal motor dysfunction, sometimes even without typical reflux symptoms (4–6). This mechanism provides biological background for the association between diabetes-related metabolic disturbance and reflux-related mucosal injury, but autonomic neuropathy is not treated as a primary analytic focus in the present study. In parallel, epidemiological and genetic evidence supports associations of GERD or erosive esophagitis with obesity, metabolic syndrome, insulin resistance, the triglyceride-glucose (TyG) index, body mass index, and T2DM (7–13). Vitamin D status has also been linked to inflammation, epithelial barrier regulation, and metabolic dysfunction, but population-level findings on 25-hydroxyvitamin D [25(OH)D] and reflux-related disease remain inconsistent (14, 15).

Although the metabolic background of reflux disease has received increasing attention, several issues remain unresolved. Prior studies have linked diabetes, autonomic dysfunction, impaired esophageal motility, obesity, metabolic syndrome, and insulin resistance with GERD or erosive esophagitis (16). Recent evidence further suggests that routine metabolic parameters may provide risk-stratification information for RE, while vitamin D status is closely related to insulin resistance, inflammatory regulation, and epithelial barrier integrity (17–19). In addition, the association between the TyG index and vitamin D deficiency in patients with T2DM suggests potential overlap between insulin-related metabolic disturbance and vitamin D status (20). However, existing evidence is not fully consistent. Some studies report positive associations between metabolic abnormalities and reflux-related disease, whereas others show weak or non-significant associations after adjustment for obesity, medication use, or other clinical factors. Findings regarding 25(OH)D are also inconsistent, and lower 25(OH)D may reflect nutritional status, adiposity, inflammatory burden, sunlight exposure, or lifestyle-related factors rather than a direct relationship with RE. Nevertheless, these findings do not fully explain the clinical-metabolic profile of endoscopy-confirmed RE in patients with established T2DM. In this paper, clinical-metabolic profile refers to the combined pattern of demographic characteristics, diabetic complications, glycemic status, vitamin D status, lipid-related indices, renal function, and insulin-related indicators. The main gaps are as follows:

1. Limited evidence based on endoscopy-confirmed RE in T2DM-specific cohorts. Most available studies are based on general health examination populations, mixed clinical cohorts, or symptom-defined reflux outcomes. Evidence focused on endoscopy-confirmed RE in established T2DM remains insufficient (21).
2. Incomplete integration of related clinical and metabolic domains. Diabetic neuropathy, glycemic control, lipid metabolism, insulin-related indices, renal function, and vitamin D status are often examined separately, although these factors frequently coexist in T2DM.
3. Unclear independence of the 25(OH)D-RE association after glycemic and insulin-related adjustment. Lower 25(OH)D may reflect inflammatory or metabolic impairment, but whether its association with RE persists after accounting for HbA1c remains uncertain (22, 23).
4. Insufficient evaluation of nonlinear metabolic patterns. HbA1c, fasting insulin, HOMA-IR, and insulin secretion index may show stronger associations at higher metabolic burden levels, but this possibility is rarely examined in previous RE studies.

The selected metabolic indicators represent related but non-identical dimensions of diabetic metabolic status. HbA1c reflects chronic glycemic exposure. The TyG index reflects combined glucose-lipid metabolic disturbance and is commonly used as a surrogate marker of insulin resistance. HOMA-IR and fasting insulin more directly reflect fasting insulin-related metabolic status, whereas fasting-state ISI represents basal insulin secretion relative to fasting glycemia in this dataset. 25(OH)D is included because vitamin D status is closely related to glycemic regulation, adiposity, low-grade inflammation, nutritional status, and lifestyle patterns, all of which may be relevant to reflux susceptibility or mucosal defense in T2DM. Evaluating these indicators within the same prespecified adjustment framework may help clarify whether the observed metabolic signal is mainly glycemic, insulin-related, vitamin D-related, or shared across these domains.

Accordingly, this study evaluates the associations of HbA1c, 25(OH)D, TyG index, HOMA-IR, fasting insulin, and fasting-state ISI with endoscopy-confirmed RE in a hospital-based T2DM population. The primary hypothesis is that patients with T2DM and endoscopy-confirmed RE have a more adverse clinical-metabolic profile than those without RE, mainly characterized by poorer glycemic control and insulin-related abnormalities. A secondary hypothesis is that lower 25(OH)D is associated with RE, but that this association attenuates after adjustment for glycemic control and insulin-related indicators. Potential nonlinear associations and exploratory risk-stratification performance of routine clinical-metabolic variables are also examined.

## 2 Methods

### 2.1 Study design and data source

This observational cross-sectional analysis uses a binary outcome framework to examine the association between clinical-metabolic characteristics and reflux esophagitis in patients with T2DM. The study period extends from July 1, 2021 to December 31, 2025. The source population comprises 11486 patients with T2DM attending the Department of Endocrinology of the Fifth Affiliated Hospital of Zhengzhou University during this period. The analytic cohort is derived from a single-center hospital-based endoscopy population within this source population. Eligible records are identified from the hospital clinical information system and laboratory database according to prespecified criteria. Patients with T2DM are first identified from clinical diagnostic records in the Department of Endocrinology, and gastrointestinal endoscopic diagnostic records are then reviewed to ascertain RE status. The final analytic cohort comprises 772 patients with ascertainable RE status and complete prespecified covariate data. Accordingly, the analytic cohort represents a hospital-based endoscopy subset of patients with T2DM rather than all patients with T2DM attending the hospital.

The primary outcome is the presence of reflux esophagitis, determined from endoscopic diagnostic records. Data are obtained from the clinical information system and laboratory database of the Fifth Affiliated Hospital of Zhengzhou University. The dataset includes demographic characteristics, anthropometric measurements, diabetes duration, comorbidities, diabetic complications, routine laboratory indicators, and endoscopic information. Records without ascertainable RE status or complete prespecified covariate data are not included in the final analytic cohort. Thus, the statement that no missing data are present applies only to the final analytic dataset after eligibility assessment, not to the full source population. RE is analyzed as a binary outcome. Although Los Angeles (LA) grade information is present in the endoscopic records, LA-A accounts for the vast majority of graded RE cases, whereas LA-B and LA-C are rare and LA-D is observed in only one patient. Therefore, LA grade is not modeled as an ordinal or severity-stratified outcome because severity-specific estimates would be statistically unstable.

The study protocol is approved by the Ethics Committee of the Fifth Affiliated Hospital of Zhengzhou University (approval no. KY2023103-K02). The requirement for written informed consent is waived by the Ethics Committee because this study uses medical records and clinical data generated during previous diagnosis and treatment, involves no direct patient contact, and requires no additional intervention. During dataset construction, direct personal identifiers are removed and each record is assigned a study code. The investigators only access the de-identified analytic dataset. The study follows the principles of the Declaration of Helsinki, and all data are used only for scientific research purposes.

### 2.2 Participants and inclusion/exclusion criteria

The analytic cohort consists of patients with T2DM who attend the Department of Endocrinology and undergo gastrointestinal endoscopy from July 1, 2021 to December 31, 2025. Eligible records are consecutively screened from the hospital clinical information system and laboratory database according to prespecified criteria. Within the source population, patients with T2DM are first identified from clinical diagnostic records, and gastrointestinal endoscopic records are then reviewed to determine RE status. Only records with ascertainable RE status and complete prespecified covariate data enter the final analytic cohort. A total of 772 patients are included, including 223 patients with RE and 549 without RE. Patients are included if they meet all of the following criteria:

1. A clear diagnosis of T2DM;
2. Available endoscopic records for determining RE status;
3. Available demographic, clinical, laboratory, endocrine, and gastrointestinal variables required for the prespecified main analyses.

Records are not included in the analytic cohort if they meet any of the following criteria:

1. Non-T2DM types of diabetes or unclear diabetes type records;
2. Missing or indeterminable RE status;
3. Incomplete prespecified clinical, laboratory, endocrine, or gastrointestinal variables after data verification;
4. Conditions that may substantially alter esophageal structure or motility, such as a history of major esophageal or gastric surgery, when identifiable in the database;
5. Identifiable conditions that may substantially influence RE assessment or metabolic status, including malignancy, chronic liver disease, connective tissue disease, or pregnancy;
6. Duplicate records or clinically implausible key values after data verification.

The complete-data requirement is applied during analytic cohort construction. Therefore, the final analytic dataset contains no missing values for variables included in the main analyses by construction, but this does not indicate absence of missingness in the full source population. Because this study is based on retrospective database extraction, criterion-specific mutually exclusive exclusion counts are not reliably reconstructable unless retained in the original screening log.

### 2.3 Definition of outcome and covariates

This study uses RE status as the dependent variable. Categorical variables are coded as 1 for yes or positive and 0 for no or negative. Continuous variables are analyzed using their original measurement values and are standardized during regression modeling when appropriate. Covariates are selected *a priori* according to clinical relevance, biological plausibility, and availability in the hospital clinical information system and laboratory database. The selected variables cover demographic characteristics, adiposity, diabetes duration, diabetes-related complications, glycemic status, insulin-related indicators, lipid parameters, renal function, vitamin D status, and gastrointestinal test findings.

#### 2.3.1 Outcome

All patients undergo routine upper gastrointestinal endoscopy. Endoscopic records are interpreted by qualified digestive endoscopists under routine departmental diagnostic standards. RE is defined by endoscopic evidence of reflux-associated mucosal injury, including visible mucosal breaks, erosion, or ulceration in the distal esophagus (8). In this paper, RE status is coded as 0 for absence and 1 for presence.

Los Angeles (LA) grade information is reviewed when recorded in the endoscopic diagnostic records. However, RE is analyzed as a binary outcome in the primary analyses. LA grade is retained for descriptive characterization only and is not included as an ordinal or severity-stratified outcome in the primary analyses, owing to the sparse distribution of higher-grade RE, including only one LA-D case. Formal inter-endoscopist agreement statistics are not captured in the retrospective database.

#### 2.3.2 Covariates

Covariates are derived from clinical registration information, past diagnostic records, and laboratory test results. These variables are included to characterize the clinical-metabolic profile of RE in patients with T2DM and to account for demographic, diabetes-related, renal, metabolic, and gastrointestinal factors associated with RE status. The main categories are as follows:

1. General Data: Gender, age, duration of diabetes, Body Mass Index (BMI).
2. Comorbidities and Diabetes Complications: Hypertension, as well as diabetic nephropathy, retinopathy, neuropathy, and vascular complications. Diabetic complications are identified from established clinical diagnoses documented in the electronic medical records and are coded as binary variables. These diagnoses are not re-adjudicated using a uniform research protocol in this retrospective analysis. Hypertension is defined as systolic blood pressure ≥140 mmHg and/or diastolic blood pressure ≥90 mmHg and is analyzed as a binary variable according to established diagnostic criteria (24).
3. Laboratory Tests: All tests are completed using venous blood collected in the morning under fasting conditions. Indicators included:

- Glucose Metabolism: Oral Glucose Tolerance Test 0 h glucose (OGTT 0H, mmol/L), Fasting Insulin (IRT 0H, μU/mL), C-peptide 0H, Glycated Hemoglobin (HbA1c, %). HbA1c is reported using the DCCT/NGSP-aligned percentage (%) system. IFCC mmol/mol values are not used in the present analyses;
- Renal Function: Blood Urea Nitrogen (BUN), Creatinine (Scr, μmol/L);
- Lipids: Total Cholesterol (TC), Triglycerides (TG), High-Density Lipoprotein Cholesterol (HDL-C), Low-Density Lipoprotein Cholesterol (LDL-C);
- Other Metabolic and Endocrine Indicators: Serum Homocysteine, 25-Hydroxyvitamin D (25(OH)D), Triiodothyronine (T3), Thyroxine (T4), Thyroid Stimulating Hormone (TSH);
- Others: Fecal occult blood and Helicobacter pylori (H. pylori) infection status are classified according to the corresponding laboratory or endoscopic test records and coded as 0 for negative and 1 for positive.
- Smoking, alcohol use, physical activity, dietary habits, medication exposure, and vitamin D supplementation are not consistently captured as structured variables in the retrospective dataset and are not included as covariates. Their potential influence is addressed in the limitation section.

(4) Derived Indices: To characterize obesity status, insulin-related metabolic burden, renal function, and metabolic syndrome, several derived indices are calculated from the original clinical and laboratory variables, including body mass index, insulin secretion index, HOMA-IR, triglyceride-glucose index, estimated glomerular filtration rate, and metabolic syndrome status. All derived variables are calculated according to prespecified formulas before model fitting. Formula-based variables are recalculated from the original clinical and laboratory measurements after unit verification.

Body mass index (BMI). BMI is calculated as weight in kilograms divided by height in meters squared:


$$BMI=\frac{Weight\;(kg)}{Height{\left(m\right)}^{2}}$$

According to Chinese adult BMI criteria, BMI is categorized as <24.0 kg/m², 24.0-27.9 kg/m², and ≥28.0 kg/m² for descriptive and stratified analyses (25).

Fasting-state insulin secretion index (ISI): In this paper, ISI refers to a fasting-state insulin-to-glucose ratio and is calculated as fasting insulin divided by fasting plasma glucose:


$$\text{ISI}=\frac{IRT\;0H}{OGTT\;0H}$$

IRT 0H denotes fasting insulin, and OGTT 0H denotes fasting plasma glucose. This index is used as a surrogate measure of basal insulin secretion relative to fasting glycemia. It does not incorporate post-load glucose, insulin, or C-peptide values and is not interpreted as a conventional dynamic insulin secretion index. The fasting-state ISI provides an available fasting-state surrogate in this retrospective dataset, as post-load insulin and C-peptide measurements are not uniformly available across the final analytic cohort. If OGTT 0H is zero or unavailable, ISI is treated as missing.

HOMA-IR. Insulin resistance is estimated using the homeostasis model assessment of insulin resistance:


$$\text{HOMA}-\text{IR}=\frac{IRT\;0H\times OGTT\;0H}{22.5}$$

Fasting insulin is expressed in μIU/mL, and fasting plasma glucose is expressed in mmol/L. A higher HOMA-IR value indicates a greater degree of insulin resistance. The HOMA-IR formula follows the original homeostasis model assessment method (26).

Triglyceride-glucose index. The triglyceride-glucose index is calculated using the conventional formula:


$$\text{TyG Index}=\text{ln}(\frac{\text{TG}(mg/dL)\times \text{FPG}(\text{mg}/\text{dL})}{2})$$

Because triglycerides and fasting plasma glucose are recorded in mmol/L, triglycerides are converted to mg/dL by multiplying by 88.57, and fasting plasma glucose is converted to mg/dL by multiplying by 18 before calculation. The TyG index formula follows established methods for this insulin-resistance surrogate marker (27).

Estimated glomerular filtration rate(eGFR): eGFR is calculated using the 2009 CKD-EPI creatinine equation, incorporating age, sex, and serum creatinine. Serum creatinine is expressed in μmol/L, and eGFR is expressed as mL/min/1.73 m².


$$eGFR=144\times \text{min}{\left(\frac{Scr}{\kappa },1\right)}^{\alpha }\times \text{max}{\left(\frac{Scr}{\kappa },1\right)}^{-1.209}\times 0.993^{Age}\times S$$

In this equation, Scr denotes serum creatinine. For men, κ=79.6, α=−0.411, and S=1. For women, κ=61.9, α=−0.329, and S=1.018. The eGFR calculation follows the 2009 CKD-EPI creatinine equation (28).

Metabolic syndrome. Metabolic syndrome is defined according to the criteria recommended by the Chinese Diabetes Society (24). Patients meeting at least three of the following four components are classified as having metabolic syndrome: obesity, glucose abnormality, hypertension, and dyslipidemia. Obesity is defined as BMI ≥25.0 kg/m² according to the Chinese Diabetes Society metabolic syndrome criteria. Glucose abnormality is defined as fasting plasma glucose ≥6.1 mmol/L, 2-hour postprandial glucose ≥7.8 mmol/L, or previously diagnosed diabetes under treatment. Hypertension is defined as systolic blood pressure ≥140 mmHg and/or diastolic blood pressure ≥90 mmHg and is analyzed as a binary variable, with the presence of hypertension coded as 1 and its absence coded as 0. Dyslipidemia is defined as triglycerides ≥1.7 mmol/L or reduced HDL-C, with HDL-C <0.9 mmol/L in men and <1.0 mmol/L in women. Because all participants have T2DM, the glucose abnormality component is considered fulfilled. Metabolic syndrome is coded as 0 for no and 1 for yes. In this T2DM cohort, metabolic syndrome represents clustering of obesity, hypertension, and dyslipidemia rather than an independent construct fully separable from diabetes. It is used as a descriptive clinical-metabolic indicator and is not treated as a primary exposure when its individual components are evaluated separately.

### 2.4 Data preprocessing and quality control

Data extraction is performed from the hospital clinical information system, laboratory database, and endoscopic diagnostic records using prespecified variable definitions. Patient identifiers are checked to remove duplicate records. Raw values are reviewed for unit consistency, impossible values, and formula compatibility. Implausible values include values with impossible signs or units, nonpositive values for variables requiring positive values in formula calculation, and values inconsistent with the corresponding original laboratory or clinical record. Extreme but clinically plausible values are retained after verification.

Formula-based variables, including BMI, HOMA-IR, TyG index, eGFR, and fasting-state ISI, are recalculated from source variables after unit conversion and verification. Derived values inconsistent with predefined formulas are corrected by recalculation from the original source measurements rather than by altering raw laboratory values. When source measurements required for calculation are unavailable or cannot be verified, the corresponding derived value is treated as missing and the record does not enter the complete-case analytic cohort. No statistical imputation is performed.

### 2.5 Statistical analysis and modeling strategy

Continuous variables are presented as medians with interquartile ranges, and categorical variables as counts and percentages. Between-group comparisons are performed using the Mann-Whitney U test for continuous variables and the chi-square test or Fisher’s exact test for categorical variables. The prevalence of reflux esophagitis is reported with a 95% confidence interval.

The analytic strategy is structured according to distinct objectives. Multivariable logistic regression models are used to evaluate independent associations between clinical-metabolic factors and RE. Substitution models assess the stability of these associations across different insulin-related indicators. Restricted cubic spline analyses examine potential nonlinear associations. Exploratory model-performance analyses evaluate risk-stratification information and are not treated as primary inferential evidence. The sequential modeling strategy is used to evaluate how the associations of selected metabolic indicators change across clinically ordered adjustment blocks, rather than to establish causal mediation.

Associations with reflux esophagitis are examined using multivariable logistic regression. To reduce data-driven model selection, covariates are entered according to prespecified adjustment blocks. Continuous variables are standardized before modeling, and odds ratios are reported per 1-standard-deviation increase. Binary variables are modeled as yes versus no. Five main models are constructed. Model 1 adjusts for age, sex, diabetes duration, and BMI. Model 2 further adjusts for hypertension, diabetic nephropathy, diabetic retinopathy, diabetic neuropathy, diabetic vascular complications, eGFR, fecal occult blood, and Helicobacter pylori status. Model 3 additionally includes 25(OH)D. Model 4 further includes HbA1c to evaluate whether glycemic control attenuates the association between 25(OH)D and reflux esophagitis. Model 5 adds TyG index to assess whether glucose-lipid metabolic burden provides additional information beyond HbA1c and 25(OH)D.

HbA1c is treated as a primary metabolic exposure in HbA1c-focused analyses and as an adjustment variable when evaluating the associations of 25(OH)D and TyG index with RE. It is not modeled as a mediator in a formal causal mediation framework. Because HbA1c, TyG index, HOMA-IR, fasting insulin, ISI, BMI, and metabolic syndrome are biologically related, attenuation after additional metabolic adjustment is interpreted as model-dependent evidence of overlapping metabolic information rather than evidence of a causal pathway. Potential overadjustment is considered when interpreting fully adjusted models.

Medication exposure, including glucose-lowering agents, proton pump inhibitors, and vitamin D supplementation, is not consistently captured as structured variables in the retrospective dataset and is therefore not included in the multivariable adjustment models. Sensitivity models are constructed by replacing TyG index in Model 5 with HOMA-IR, fasting insulin, or ISI. Multicollinearity is assessed using variance inflation factors for predictors included in each multivariable model and sensitivity model, with values >5 considered to indicate potentially meaningful multicollinearity. Detailed results are presented in **Supplementary Table 7**.

Additional categorical analyses use prespecified clinically interpretable categories. HbA1c is categorized as <7%, 7% to <9%, and ≥9% for exploratory clinical interpretation. Vitamin D status is categorized as deficient [25(OH)D <20 ng/mL], insufficient [20 to <30 ng/mL], and sufficient [≥30 ng/mL], with the sufficient group as the reference category. BMI is categorized according to Chinese adult BMI criteria as <24.0 kg/m², 24.0-27.9 kg/m², and ≥28.0 kg/m² (25). Hypertension is defined as systolic blood pressure ≥140 mmHg and/or diastolic blood pressure ≥90 mmHg, consistent with the hypertension component of the Chinese Diabetes Society metabolic syndrome criteria (24). Metabolic syndrome is defined according to the criteria recommended by the Chinese Diabetes Society, as detailed in Section 2.3.2 (24). HOMA-IR, TyG index, eGFR, and fasting-state ISI are calculated according to prespecified formulas and are analyzed as continuous variables unless otherwise stated (26–28).

Univariate logistic regression is retained as an exploratory analysis, and false discovery rate correction is applied using the Benjamini-Hochberg method. Exploratory model-performance analyses, including area under the receiver operating characteristic curve, average precision, Brier score, and calibration metrics, are performed as secondary analyses because no external validation cohort is available. These analyses are used only to assess the risk-stratification information provided by routine clinical-metabolic variables and are not intended to develop a clinically deployable prediction model. Model performance is evaluated using the area under the receiver operating characteristic curve, average precision, Brier score, calibration intercept, calibration slope, calibration curves, and the Hosmer-Lemeshow goodness-of-fit test. Internal validation is performed using 10-fold cross-validation.

The augmented model is constructed by adding HbA1c and 25(OH)D to the clinical-metabolic model containing demographic characteristics, diabetes-related complications, renal function, gastrointestinal test findings, and TyG index. Predicted probabilities are generated from logistic regression models within each validation fold, and discrimination and calibration metrics are summarized across validation folds. All statistical tests are two-sided, with P<0.05 considered statistically significant.

## 3 Results

### 3.1 Cohort derivation and RE prevalence

**Figure 1:**
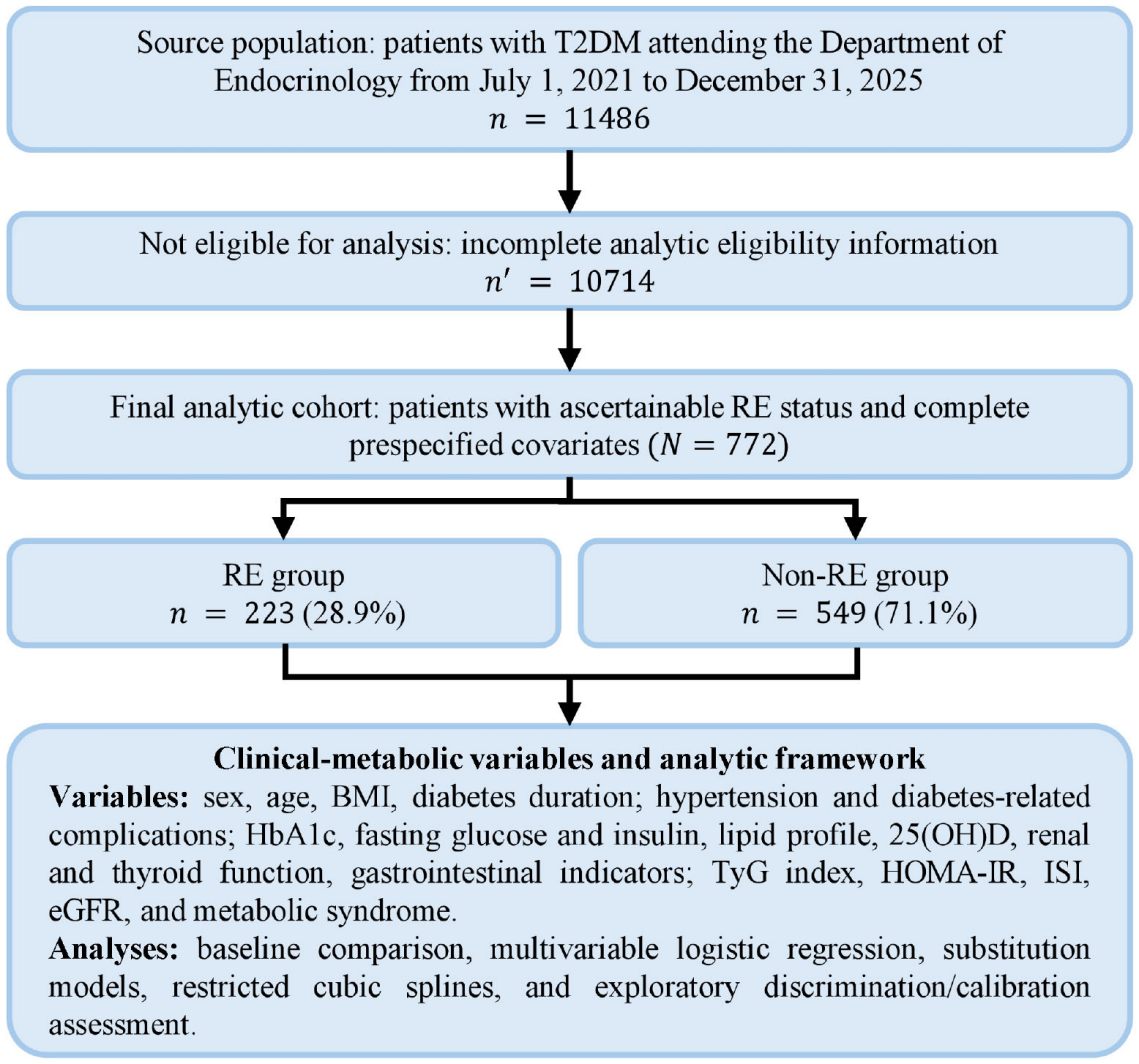
Cohort derivation and analytic framework.

A total of 11,486 patients with T2DM attending the Department of Endocrinology from July 1, 2021 to December 31, 2025 are identified as the source population. After analytic eligibility assessment, 772 patients with ascertainable RE status from endoscopic records and complete prespecified covariates are included in the final analytic cohort. A total of 10,714 patients are not included because analytic eligibility criteria are not fulfilled. Based on endoscopic diagnostic records, 223 patients are classified into the RE group and 549 into the non-RE group. The observed proportion of RE in the analytic cohort is 28.9% (223/772), with a 95% confidence interval of 25.8%-32.2%. This estimate is based on hospital-based patients with T2DM who had ascertainable endoscopic RE status and complete prespecified covariate data, and should not be interpreted as the population prevalence of RE among all patients with T2DM. The cohort derivation and analytic framework are presented in **Figure 1**.

### 3.2 Baseline characteristic comparison

Baseline characteristics according to reflux esophagitis status are summarized in **Table 1**. Patients with RE have a higher proportion of men, slightly older age, and higher BMI than those without RE. Hypertension, diabetic neuropathy, and metabolic syndrome are also more frequent in the RE group. For metabolic indicators, patients with RE show higher HbA1c, fasting insulin, triglycerides, HOMA-IR, insulin secretion index, and TyG index. Serum 25(OH)D is lower in the RE group. No marked between-group differences are observed in diabetes duration, fasting plasma glucose, renal function, uric acid, thyroid function, fecal occult blood positivity, or Helicobacter pylori status. The absolute between-group differences for several statistically significant variables are modest and are therefore interpreted in conjunction with effect sizes in the regression analyses. Baseline standardized mean differences are provided in **Supplementary Figure 1**.

**Table 1:** Baseline characteristics according to reflux esophagitis status.

Characteristic	Overall (N = 772)	Non-RE (n=549)	RE (n=223)	P value
Demographics and anthropometrics				
Male, n (%)	462 (59.8)	315 (57.4)	147 (65.9)	0.028
Age (years)	60 (54-67)	59 (53-67)	61 (55-68)	0.039
Diabetes duration (years)	8 (2.75-15)	8 (3-15)	7 (2-15)	0.720
BMI (kg/m²)	24.61 (22.86-26.96)	24.48 (22.75-26.78)	24.91 (23.16-27.44)	<0.01
Comorbidities and diabetic complications				
Hypertension, n (%)	461 (59.7)	315 (57.4)	146 (65.5)	0.038
Metabolic syndrome, n (%)	399 (51.7)	267 (48.6)	132 (59.2)	<0.01
Diabetic nephropathy, n (%)	323 (41.8)	231 (42.1)	92 (41.3)	0.834
Diabetic retinopathy, n (%)	120 (15.5)	85 (15.5)	35 (15.7)	0.941
Diabetic neuropathy, n (%)	511 (66.2)	351 (63.9)	160 (71.7)	0.038
Vascular complications, n (%)	643 (83.3)	457 (83.2)	186 (83.4)	0.955
Glycemic and insulin-related measures				
Fasting plasma glucose (mmol/L)	7.08 (6.66-7.67)	7.07 (6.63-7.70)	7.09 (6.71-7.66)	0.966
HbA1c (%)	8.55 (6.6-10.2)	8.0 (6.6-10)	9.4 (7.8-11.7)	<0.01
Fasting insulin (μIU/mL)	8.23 (6.25-12.39)	8.17 (6.15-10.83)	8.58 (7.32-15.16)	<0.01
Fasting C-peptide (ng/mL)	1.5 (1.29-1.89)	1.5 (1.29-1.9)	1.51 (1.31-1.87)	0.956
HOMA-IR	2.62 (1.92-3.89)	2.59 (1.87-3.53)	2.78 (2.1-5.3)	<0.010
Insulin secretion index (ISI)	1.16 (0.89-1.64)	1.15 (0.87-1.47)	1.26 (0.99-2.22)	<0.01
TyG index	9.16 (8.70-9.54)	9.14 (8.68-9.50)	9.21 (8.80-9.68)	0.042
Renal function and lipid profile				
Blood urea nitrogen (mmol/L)	5.7 (4.88-6.8)	5.7 (4.9-6.8)	5.7 (4.65-6.9)	0.650
Creatinine (μmol/L)	74 (57-94)	74 (53-94)	75 (59-96)	0.477
eGFR (mL/min/1.73 m²)	91.30 (67.27-103.73)	91.77 (67.17-105.01)	89.98 (68.02-101.21)	0.384
Total cholesterol (mmol/L)	4.46 (3.91-5.2)	4.47 (3.92-5.2)	4.45 (3.88-5.17)	0.520
Triglycerides (mmol/L)	1.6 (1.08-2.46)	1.54 (1.05-2.35)	1.71 (1.17-2.78)	0.021
HDL-C (mmol/L)	1.25 (1.05-1.51)	1.25 (1.05-1.5)	1.27 (1.04-1.53)	0.623
LDL-C (mmol/L)	2.37 (1.84-3.01)	2.37 (1.84-3.03)	2.36 (1.83-2.96)	0.461
Other laboratory and gastrointestinal measures				
Homocysteine (μmol/L)	13.1 (11-15.6)	13.1 (11-15.5)	13.1 (10.7-15.8)	0.718
25(OH)D (ng/mL)	17.2214.04-22.49)	17.40 (14.20-23.02)	3	0.038
T3	4.83 (4.45-5.25)	4.82 (4.42-5.22)	4.9 (4.46-5.29)	0.420
T4	15.1 (14.05-16.76)	15.12 (14.03-16.81)	15.08 (14.08-16.55)	0.438
TSH	2.02 (1.31-2.91)	2.02 (1.29-2.91)	2.03 (1.43-2.92)	0.656
Fecal occult blood positive, n (%)	122 (15.8)	83 (15.1)	39 (17.5)	0.413
Helicobacter pylori positive, n (%)	197 (25.5)	139 (25.3)	58 (26.0)	0.842

### 3.3 Univariate association analysis

Exploratory univariate logistic regression analyses are performed to describe the crude association pattern between candidate variables and RE. Continuous variables are standardized and reported per 1-standard-deviation increase, whereas binary variables are modeled as yes versus no. HbA1c shows the strongest crude association with RE. Insulin-related indicators, including fasting insulin, insulin secretion index, and HOMA-IR, also show positive crude associations after false discovery rate correction. TyG index, metabolic syndrome, BMI, male sex, hypertension, diabetic neuropathy, and older age show nominal positive associations with RE. Serum 25(OH)D shows an inverse crude association.

**Figure 2:**
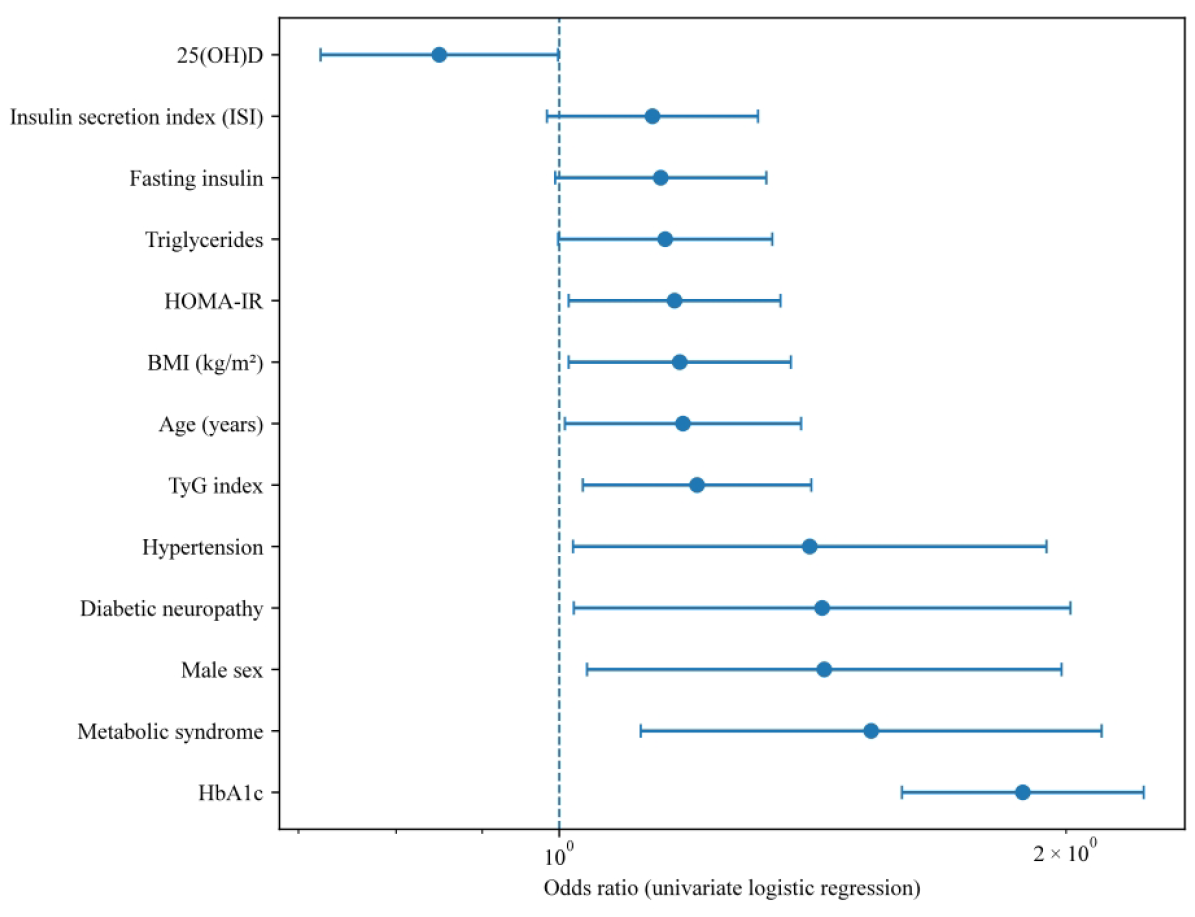
Exploratory univariate associations with reflux esophagitis.

Selected univariate estimates are summarized in **Table 2** and **Figure 2**. Because these analyses are exploratory and involve multiple comparisons, the results are interpreted descriptively and are not used as the sole basis for variable selection in the primary multivariable models. Full univariate results with false discovery rate correction are provided in **Supplementary Table 1**, and clinically interpretable estimates for selected continuous predictors are provided in **Supplementary Table 2**.

**Table 2:** Selected exploratory univariate associations with reflux esophagitis.

Predictor	aOR (95% CI)	P value	FDR q value
HbA1c	1.55 (1.33-1.81)	<0.001	<0.001
Fasting insulin	1.28 (1.10-1.49)	0.008	0.025
Insulin secretion index	1.26 (1.09-1.47)	0.002	0.025
HOMA-IR	1.27 (1.08-1.48)	0.003	0.027
Metabolic syndrome	1.53 (1.12-2.10)	0.008	0.051
25(OH)D	0.81 (0.69-0.96)	0.014	0.077
Male sex	1.44 (1.04-1.99)	0.029	0.107
TyG index	1.19 (1.02-1.39)	0.030	0.107
BMI	1.18 (1.01-1.37)	0.033	0.107
Diabetic neuropathy	1.43 (1.02-2.01)	0.038	0.107
Hypertension	1.41 (1.02-1.95)	0.038	0.107
Age	1.18 (1.01-1.39)	0.040	0.107

### 3.4 Multivariate logistic regression analysis

Multivariable logistic regression models are constructed using prespecified adjustment blocks to evaluate the adjusted associations between metabolic indicators and RE. Continuous variables are standardized and reported per 1-standard-deviation increase, and binary variables are modeled as yes versus no. The main model results are summarized in **Table 3**, with full model estimates provided in **Supplementary Table 3**.

**Table 3:** Prespecified multivariable logistic regression models for reflux esophagitis.

Predictor	Model 1	Model 2	Model 3	Model 4	Model 5
Male sex	1.45 (1.04-2.01)	1.44 (1.01-2.06)	1.53 (1.07-2.19)	1.52 (1.05-2.19)	1.51 (1.04-2.19)
Age	1.24 (1.05-1.46)	1.22 (1.01-1.48)	1.22 (1.01-1.48)	1.24 (1.03-1.50)	1.24 (1.03-1.50)
Diabetes duration	0.96 (0.82-1.14)	0.95 (0.78-1.15)	0.94 (0.77-1.14)	0.98 (0.80-1.20)	0.98 (0.80-1.20)
BMI	1.21 (1.03-1.43)	1.16 (0.98-1.37)	1.15 (0.97-1.36)	1.12 (0.95-1.33)	1.12 (0.94-1.33)
25(OH)D	–	–	0.80 (0.68-0.96)	0.84 (0.70-1.00)	0.84 (0.70-1.00)
HbA1c	–	–	–	1.58 (1.35-1.86)	1.58 (1.33-1.87)
TyG index	–	–	–	–	1.02 (0.85-1.21)

In Model 1, male sex, older age, and higher BMI are positively associated with RE. After additional adjustment for comorbidities, diabetic complications, eGFR, fecal occult blood positivity, and HP status in Model 2, male sex and age remain nominally associated with RE, whereas the association for BMI is attenuated. When 25(OH)D is added in Model 3, higher 25(OH)D is associated with lower odds of RE. After HbA1c is further included in Model 4, the association between 25(OH)D and RE is attenuated and becomes borderline, while HbA1c shows a strong positive association with RE. In Model 5, further inclusion of the TyG index does not materially change the association of HbA1c with RE, and the TyG index does not show an additional adjusted association. Across these cross-sectional models, HbA1c shows the most consistent adjusted association with RE. The inverse association between 25(OH)D and RE is evident before HbA1c adjustment but weakens after accounting for glycemic status. Male sex and age remain positively associated with RE across the main models, whereas the association for BMI attenuates after additional clinical and metabolic adjustment.

**Figure 3:**
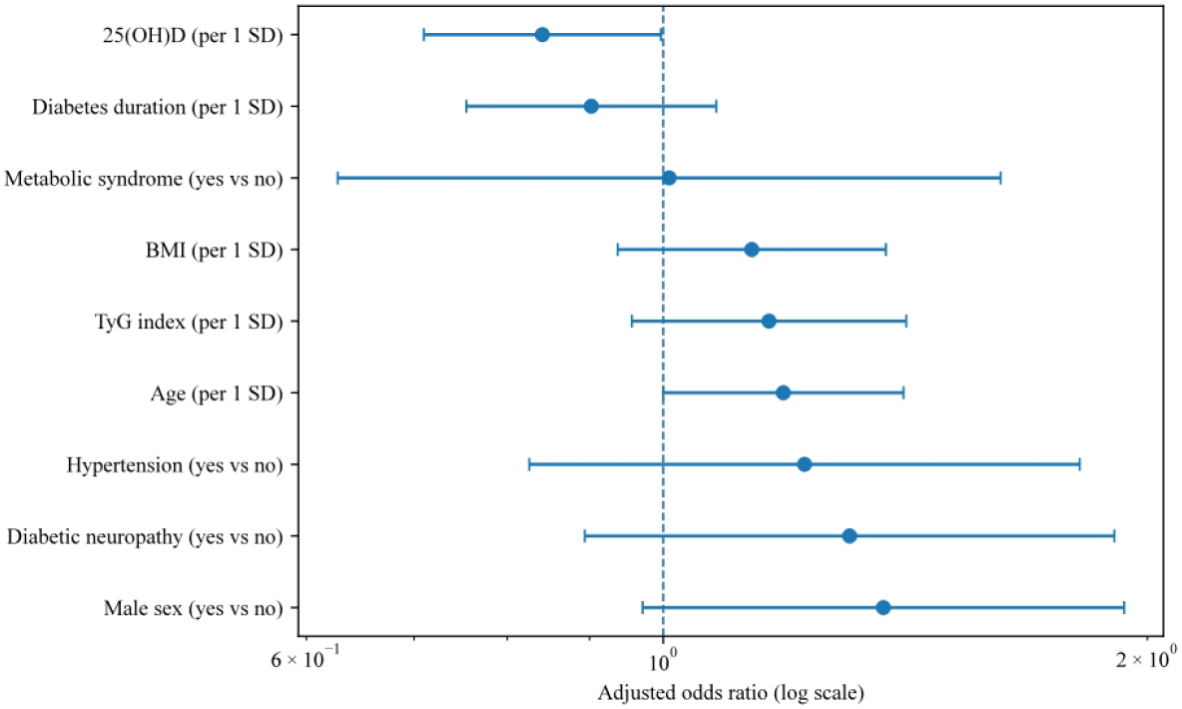
Adjusted associations of key metabolic indicators with reflux esophagitis across prespecified models.

To provide a more clinically interpretable assessment of vitamin D status, an additional categorical analysis is performed using deficient, insufficient, and sufficient 25(OH)D groups. RE prevalence is 31.5% in the deficient group, 24.9% in the insufficient group, and 18.4% in the sufficient group. Compared with the sufficient group, the insufficient group shows no statistically significant association with RE in the fully adjusted model (OR = 1.37, 95% CI: 0.60-3.12; P = 0.452). The deficient group shows a higher point estimate, but the association is not statistically significant after full adjustment (OR = 1.76, 95% CI: 0.80-3.86; P = 0.156). The ordinal trend across worsening vitamin D categories is also attenuated after adjustment for HbA1c and TyG index (P for trend=0.075). The full categorical vitamin D analysis is provided in **Supplementary Table 6**. The adjusted associations of key metabolic indicators across Models 3–5 are shown in **Figure 3**. Continuous variables are standardized and reported per 1-standard-deviation increase. Model 3 includes 25(OH)D after adjustment for demographic and clinical covariates; Model 4 further includes HbA1c; Model 5 further includes the TyG index.

Given the correlation among insulin-related indicators, additional sensitivity models are constructed by replacing the TyG index in Model 5 with HOMA-IR, fasting insulin, and insulin secretion index. The correlation structure of major continuous metabolic variables is provided in **Supplementary Figure 2**. As shown in **Table 4**, HbA1c remains consistently associated with RE across all substitution models. HOMA-IR, fasting insulin, and insulin secretion index show positive adjusted associations when entered separately. In contrast, the TyG index does not provide additional information after adjustment for HbA1c and 25(OH)D. The inverse direction of the 25(OH)D association is generally consistent across the substitution models, although its statistical strength varies. Alternative multivariable models using the TyG index as the insulin-resistance surrogate are provided in **Supplementary Table 4**. Across the main multivariable models and sensitivity models, variance inflation factors range from 1.01 to 1.53, and all values are below the prespecified threshold of 5. These findings indicate no evidence of meaningful multicollinearity among the included predictors. Detailed results are presented in **Supplementary Table 7**.

**Table 4:** Sensitivity analyses using alternative insulin-related indicators.

Insulin-related indicator	25(OH)D aOR (95% CI)	HbA1c aOR (95% CI)	Indicator aOR (95% CI)
TyG index	0.84 (0.70-1.00)	1.58 (1.33-1.87)	1.02 (0.85-1.21)
HOMA-IR	0.82 (0.69-0.98)	1.55 (1.32-1.83)	1.23 (1.03-1.46)
Fasting insulin	0.82 (0.69-0.98)	1.56 (1.33-1.84)	1.26 (1.06-1.50)
Insulin secretion index	0.83 (0.70-0.99)	1.57 (1.34-1.86)	1.26 (1.07-1.49)

### 3.5 Exploratory model-performance analysis

#### 3.5.1 Discrimination performance

Exploratory discrimination analyses are performed to assess whether routine clinical and metabolic variables provide additional risk-stratification information for RE. These analyses are considered secondary because the primary aim of this study is to identify metabolic correlates rather than to develop a clinical prediction model.

**Figure 4:**
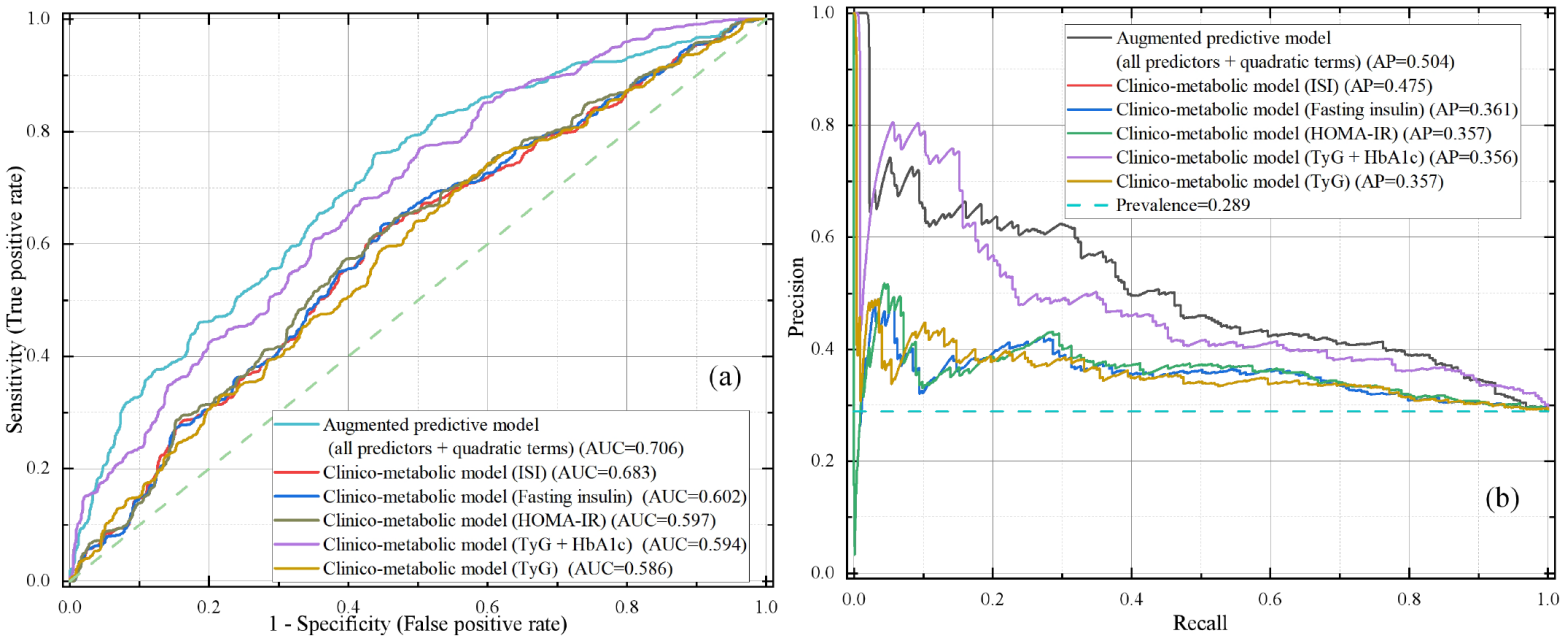
Discrimination performance based on OOF predicted probabilities from stratified 3-fold cross-validation. **(a)** ROC curves and **(b)** PR curves.

The clinical-metabolic model based on the TyG index shows limited discrimination, with a mean AUC of 0.579 and a mean average precision of 0.385. After HbA1c is added, model performance improves, with the mean AUC increasing to 0.680 and the mean average precision increasing to 0.493. Models using HOMA-IR, fasting insulin, or fasting-state ISI show similar limited discrimination. The augmented exploratory model shows the highest exploratory performance, with a mean AUC of 0.688, a mean average precision of 0.500, and a mean Brier score of 0.190. Overall, discrimination remains modest across all models. These findings indicate that routine clinical-metabolic variables provide limited risk-stratification information, and the models are not suitable for clinical decision-making without external validation. The ROC and precision-recall curves are shown in **Figure 4**, and the corresponding performance metrics are summarized in **Table 5**.

**Table 5:** Discrimination and overall performance based on stratified 10-fold cross-validation.

Model	AUC (mean ± SD)	AP (mean ±SD)	Brier score (mean ± SD)
Clinico-metabolic model (TyG)	0.579 ± 0.074	0.385 ± 0.082	0.204 ± 0.010
Clinico-metabolic model (TyG + HbA1c)	0.680 ± 0.067	0.493 ± 0.082	0.191 ± 0.014
Clinico-metabolic model (HOMA-IR)	0.589 ± 0.053	0.384 ± 0.064	0.204 ± 0.009
Clinico-metabolic model (Fasting insulin)	0.585 ± 0.055	0.379 ± 0.067	0.204 ± 0.009
Clinico-metabolic model (ISI)	0.583 ± 0.055	0.375 ± 0.067	0.204 ± 0.008
Augmented exploratory model	0.688 ± 0.043	0.500 ± 0.072	0.190 ± 0.015

#### 3.5.2 Calibration performance

**Figure 5:**
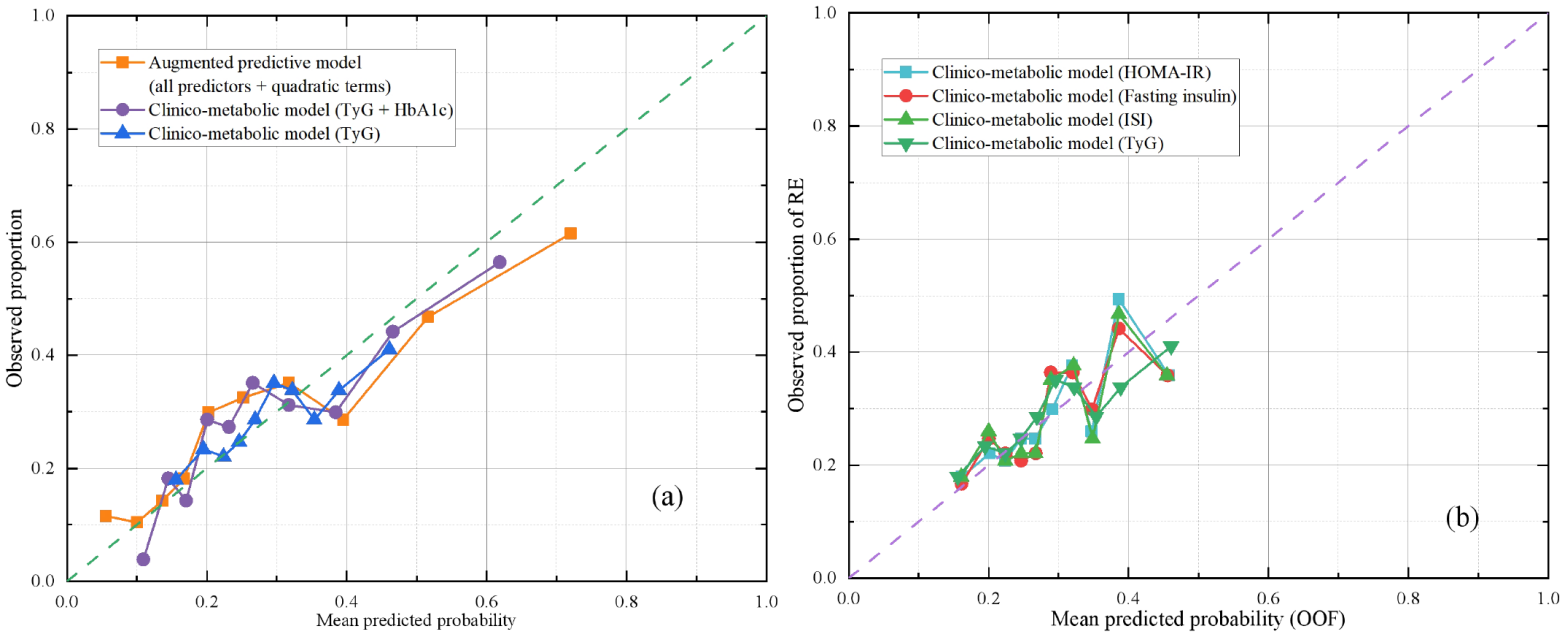
Calibration curves for key and sensitivity model. **(a)** key models; **(b)** sensitivity models.

Calibration performance is assessed separately from discrimination performance using grouped calibration curves, calibration intercept, calibration slope, and the Hosmer-Lemeshow goodness-of-fit test. As shown in **Figure 5** and **Table 6**, the calibration curves indicate that predicted probabilities do not perfectly align with observed RE proportions across all risk strata. The augmented exploratory model shows the lowest Brier score, but calibration remains imperfect. The Hosmer-Lemeshow test indicates evidence of miscalibration for the TyG + HbA1c model and the augmented exploratory model, with P values of 0.043 and 0.006, respectively. Predicted probabilities from these models should therefore not be interpreted as reliable individualized risk estimates in clinical practice. The calibration results support the exploratory use of these models only.

**Table 6:** Calibration performance and Hosmer-Lemeshow goodness-of-fit test.

Model	Calibration intercept	Calibration slope	HL χ²	df	P value
Clinico-metabolic model (TyG)	-0.275	0.695	5.593	8	0.693
Clinico-metabolic model (TyG + HbA1c)	-0.125	0.863	15.926	8	0.043
Clinico-metabolic model (HOMA-IR)	-0.211	0.765	11.229	8	0.189
Clinico-metabolic model (Fasting insulin)	-0.255	0.715	10.046	8	0.262
Clinico-metabolic model (ISI)	-0.270	0.697	14.239	8	0.076
Augmented predictive model	-0.224	0.707	21.268	8	0.006

**Figure 6:**
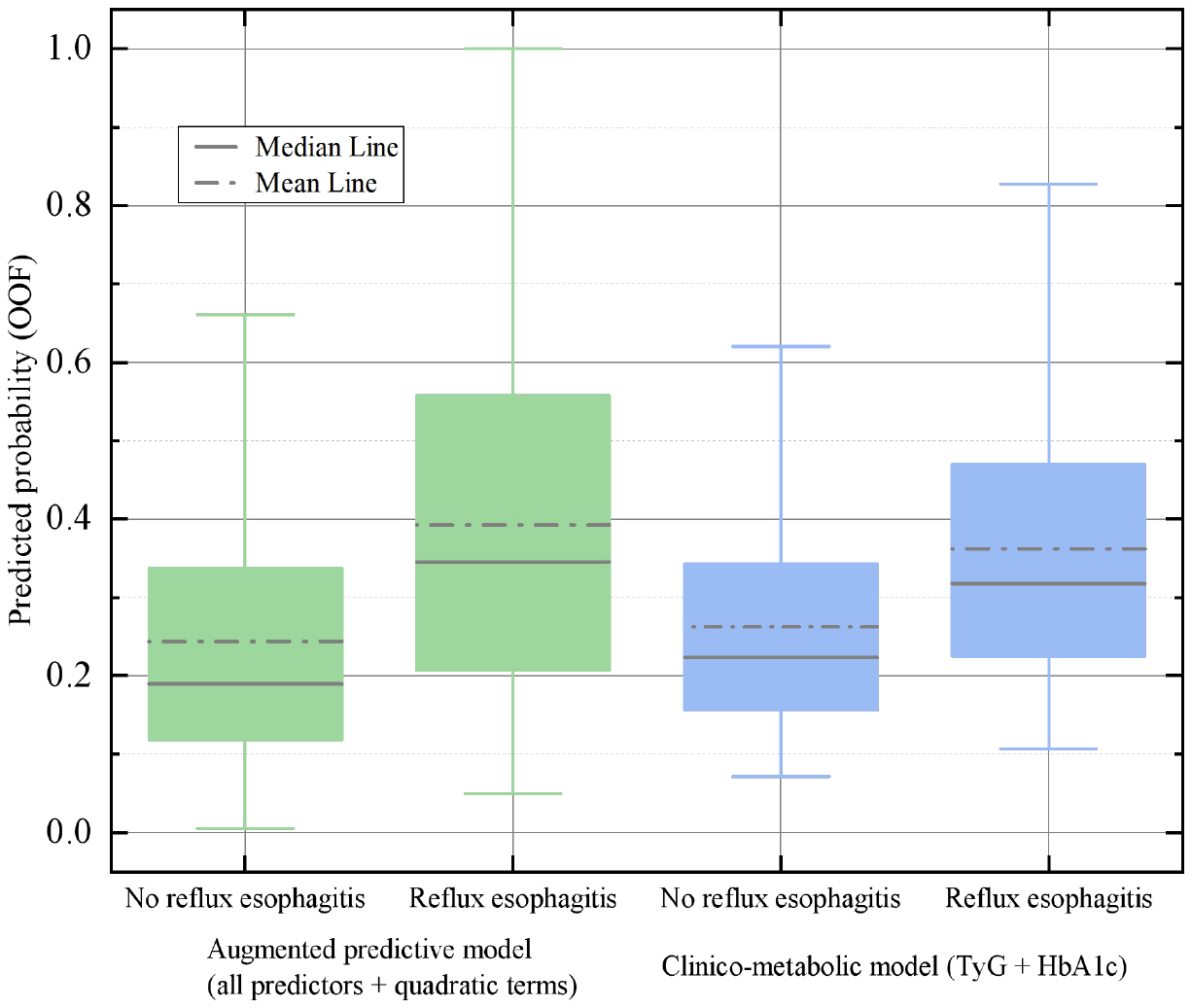
Predicted risk distribution of RE and non-RE populations.

The distributions of predicted probabilities are shown in **Figure 6**. The TyG-based model shows substantial overlap between RE and non-RE patients. After HbA1c is added, the separation between the two groups becomes more apparent, although considerable overlap remains. This pattern is consistent with the moderate discrimination observed in the ROC and precision-recall analyses. The corresponding robustness metrics based on 10-fold cross-validation are summarized in **Table 7**.

**Table 7:** 10-fold CV mean ± SD (robustness).

Model	AUC (mean ± SD)	AP (mean ± SD)	Brier score (mean ± SD)
Clinico-metabolic model (TyG)	0.579 ± 0.074	0.385 ± 0.082	0.204 ± 0.010
Clinico-metabolic model (TyG + HbA1c)	0.680 ± 0.067	0.493 ± 0.082	0.191 ± 0.014
Clinico-metabolic model (HOMA-IR)	0.589 ± 0.053	0.384 ± 0.064	0.204 ± 0.009
Clinico-metabolic model (Fasting insulin)	0.585 ± 0.055	0.379 ± 0.067	0.204 ± 0.009
Clinico-metabolic model (ISI)	0.583 ± 0.055	0.375 ± 0.067	0.204 ± 0.008
Augmented predictive model (all predictors + quadratic terms)	0.688 ± 0.043	0.500 ± 0.072	0.190 ± 0.015

### 3.6 Non-linear relationships of key continuous indicators

**Figure 7:**
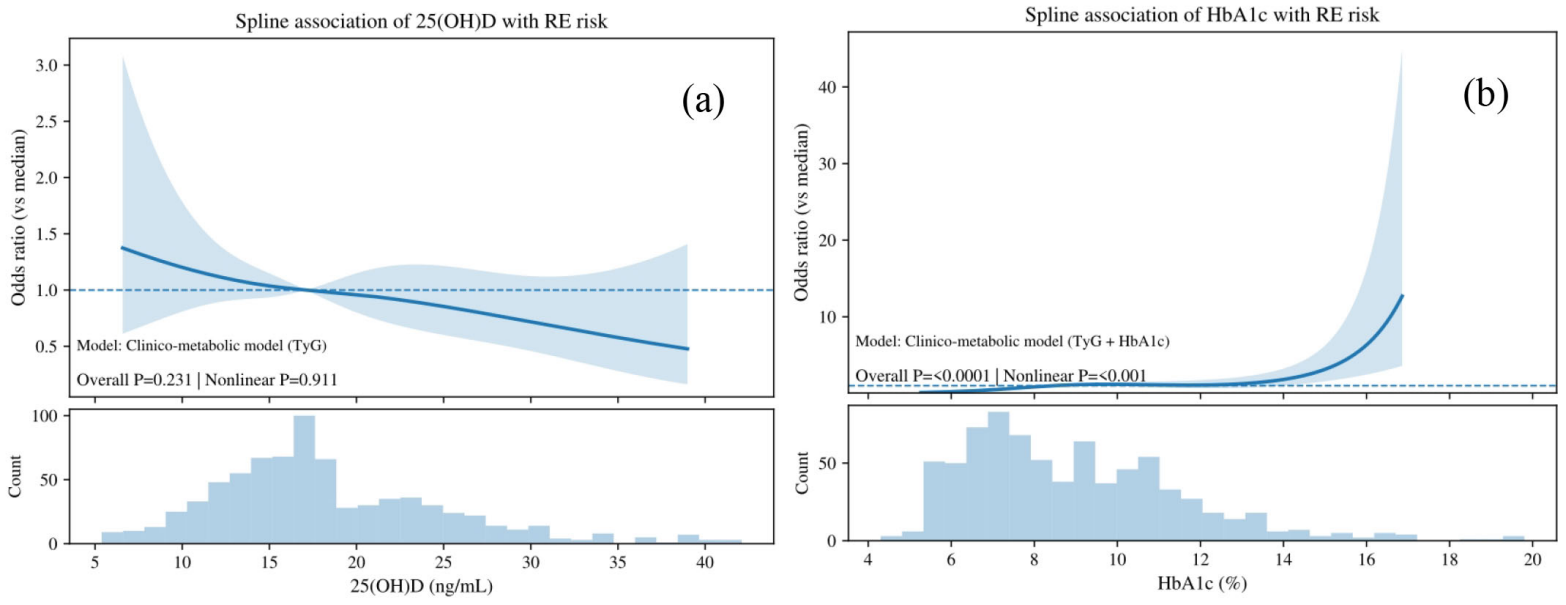
Restricted cubic spline associations of 25(OH)D and HbA1c with RE risk. **(a)** 25(OH)D; **(b)** HbA1c.

Restricted cubic spline analyses are used to examine potential nonlinear associations between selected continuous indicators and RE, with odds ratios estimated relative to the median value of each exposure. The main analyses focus on 25(OH)D and HbA1c, followed by insulin-related indicators, age, and BMI. As shown in **Figure 7** and **Table 8**, 25(OH)D shows an inverse trend with RE, but the overall association does not reach statistical significance and there is no evidence of nonlinearity (overall P = 0.088; nonlinear P = 0.913). The confidence intervals widen at both ends of the curve, indicating greater uncertainty in extreme value ranges. In contrast, HbA1c shows a significant overall association with RE and a significant nonlinear component (overall P<0.001; nonlinear P<0.001), with the spline curve indicating stronger associations at higher HbA1c levels.

**Table 8:** Summary of restricted cubic spline tests and effect estimates for key continuous predictors.

Variable	Knots (P5/P35/P65/P95)	Reference (median)	Overall P	Nonlinear P	P10 value	OR at P10 vs median (95% CI)	P90 value	OR at P90 vs median (95% CI)
25(OH)D	9.52, 15.37, 19.17, 30.61	17.100	0.231	0.911	11.105	1.16 (0.86-1.55)	27.008	0.80 (0.55-1.18)
HbA1c	5.70, 7.40, 9.70, 13.60	8.450	<0.0001	<0.001	6.100	0.23 (0.13-0.39)	12.200	1.09 (0.66-1.79)
TyG index	8.10, 8.92, 9.38, 10.38	9.158	0.293	0.968	8.271	0.83 (0.56-1.24)	10.112	1.26 (0.90-1.77)
HOMA-IR	0.95, 2.40, 3.06, 10.52	2.615	<0.0001	<0.0001	1.147	0.84 (0.54-1.31)	6.932	2.33 (1.63-3.33)
Fasting insulin	3.23, 7.76, 8.98, 28.52	8.230	<0.0001	<0.0001	4.097	0.77 (0.51-1.17)	21.037	2.60 (1.79-3.76)
ISI	0.43, 1.06, 1.36, 4.07	1.164	<0.0001	<0.0001	0.548	0.81 (0.54-1.23)	2.692	2.47 (1.75-3.47)
Age	37.00, 56.00, 64.00, 75.00	60.000	0.107	0.336	46.000	0.73 (0.46-1.16)	72.000	1.02 (0.75-1.37)
BMI	19.64, 23.59, 25.74, 31.45	24.605	0.329	0.412	20.671	0.88 (0.63-1.22)	29.127	1.46 (0.97-2.21)

**Figure 8:**
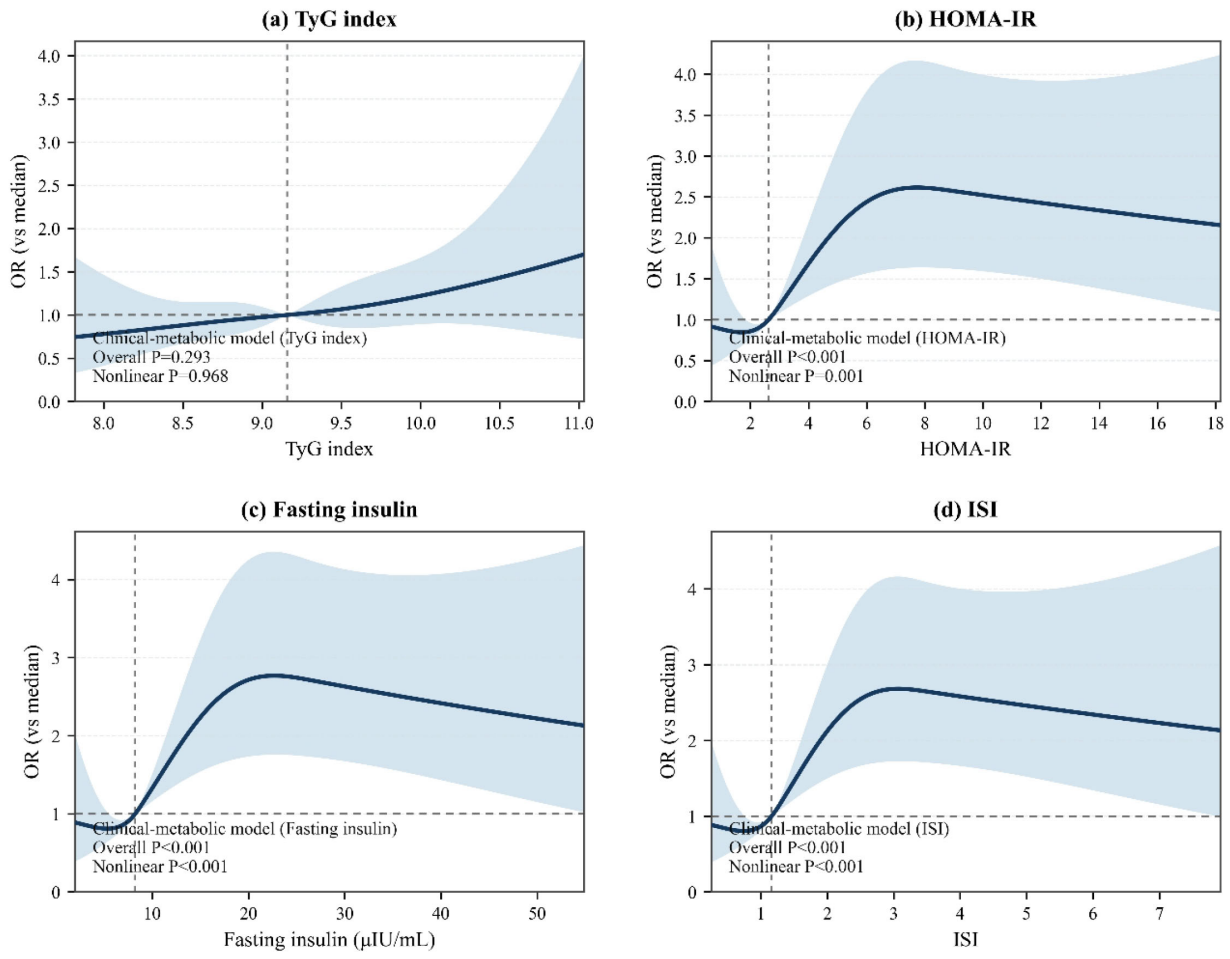
Restricted cubic spline associations of insulin-related indicators with RE risk. **(a)** TyG; **(b)** HOMA-IR; **(c)** Fasting insulin; **(d)** ISI.

Additional spline analyses for insulin-related indicators are shown in **Figure 8**. HOMA-IR, fasting insulin, and insulin secretion index show significant overall and nonlinear associations with RE. Higher values of these indicators are associated with higher odds of RE in the upper range of their distributions. In contrast, the TyG index does not show a significant overall association or clear evidence of nonlinearity in the spline framework, consistent with its limited adjusted contribution in the multivariable models.

**Figure 9:**
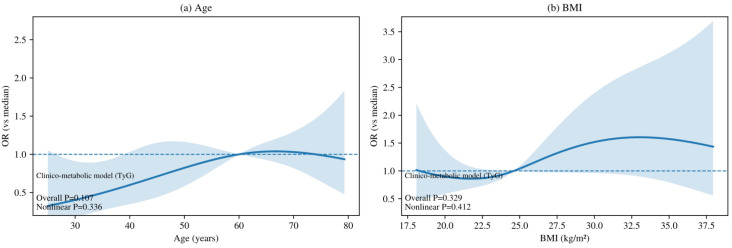
Restricted cubic spline associations of age and BMI with reflux esophagitis. **(A)** Age; **(B)** body mass index (BMI).

Age and BMI are further examined in spline analyses. As shown in **Figure 9**, neither variable demonstrates a strong nonlinear pattern in the adjusted spline framework. The spline analyses therefore identify the most evident nonlinear patterns for HbA1c and selected insulin-related indicators, whereas 25(OH)D shows an approximately linear inverse trend with wider uncertainty at extreme values.

Overall, the spline analyses support three observations. First, HbA1c shows the clearest non-linear association with RE, with higher odds concentrated at elevated HbA1c levels. Second, insulin-related indicators show similar non-linear patterns, particularly in higher ranges. Third, 25(OH)D shows an inverse but approximately linear trend, with greater uncertainty at extreme values. These findings are consistent with the main regression analyses and further support glycemic control as a central metabolic correlate of RE.

## 4 Discussion

### 4.1 Principal findings

This real-world cross-sectional study evaluates the clinical and metabolic correlates of reflux esophagitis in patients with T2DM. The analysis yields several observations: First, RE is common in this population, with an observed prevalence of 28.9%. Second, patients with RE show a more adverse metabolic profile, characterized by higher HbA1c, higher fasting insulin, higher triglycerides, higher insulin-related indices, and lower 25(OH)D levels. Third, in prespecified multivariable models, HbA1c shows the most consistent association with RE. The inverse association between 25(OH)D and RE is evident before HbA1c adjustment but attenuates after glycemic control is included. Fourth, insulin-related indicators, particularly fasting insulin, HOMA-IR, and insulin secretion index, provide additional metabolic information, whereas the TyG index shows limited incremental value after HbA1c adjustment. Exploratory discrimination analyses further suggest that routine clinical-metabolic variables provide limited to moderate risk-stratification capacity. Although several baseline characteristics differ between patients with and without RE, some absolute between-group differences are modest. These differences should therefore be interpreted cautiously and in conjunction with adjusted effect estimates, confidence intervals, and the cross-sectional design.

In relation to the broader literature, the present findings are generally consistent with systematic reviews, meta-analyses, and epidemiological studies suggesting links among diabetes, metabolic dysfunction, autonomic abnormalities, and reflux-related disease. Diabetic autonomic neuropathy has been reported to affect gastrointestinal function, including delayed esophageal transit and gastroparesis, providing a plausible neurofunctional pathway linking diabetes with reflux exposure and impaired esophageal clearance (29). Functional studies using impedance-pH monitoring, esophageal manometry, heart rate variability assessment, and high-resolution manometry have also suggested that patients with diabetes may have abnormal reflux burden or esophageal motor dysfunction, sometimes even without typical reflux symptoms (4–6). In parallel, previous epidemiological evidence has associated GERD or erosive esophagitis with obesity, metabolic syndrome, insulin resistance, the TyG index, BMI, and T2DM (7–13, 16, 22, 23). These findings support the interpretation of RE in T2DM as part of a broader metabolic and neurofunctional phenotype rather than an isolated gastrointestinal condition. However, the literature is not uniform. Some studies have reported weak or non-significant associations after adjustment for obesity, medication exposure, or other clinical factors, and findings regarding 25(OH)D and reflux-related outcomes remain inconsistent. Therefore, the present results should be interpreted as evidence from a hospital-based T2DM cohort with endoscopy-confirmed RE, rather than as definitive evidence of a universal metabolic pathway.

### 4.2 25(OH)D, glycemic control, and reflux esophagitis

The observed inverse association between 25(OH)D and RE is biologically plausible but should be interpreted cautiously. Vitamin D status may relate to mucosal immune regulation, epithelial barrier function, systemic inflammation, and metabolic health. Recent evidence suggests that vitamin D signaling may contribute to gastrointestinal homeostasis through regulation of inflammation, tight junctions, and intestinal epithelial barrier function (30). Lower 25(OH)D levels often coexist with obesity, insulin resistance, reduced outdoor activity, renal dysfunction, poor nutritional status, and chronic low-grade inflammation. In patients with T2DM, vitamin D status has also been linked to metabolic control and oxidative stress-related markers, although the metabolic effects of supplementation remain inconsistent (31). These factors may also influence reflux exposure or esophageal mucosal vulnerability. Seasonal variation in 25(OH)D measurements may further contribute to between-patient differences in vitamin D status, and season of measurement is not adjusted for in this analysis.

Importantly, the association between 25(OH)D and RE weakens after HbA1c adjustment. This pattern suggests that lower 25(OH)D may be a marker of broader metabolic or behavioral factors rather than an independent protective factor. The categorical analysis based on deficient, insufficient, and sufficient 25(OH)D groups provides a more clinically interpretable assessment of this association. Although the observed proportion of RE increases with worsening vitamin D status, the fully adjusted categorical analyses do not provide definitive evidence of an independent threshold-based or graded association. However, the cross-sectional design cannot distinguish metabolic impairment from unmeasured lifestyle confounding, seasonal variation, nutritional status, or differences in outdoor activity and sunlight exposure. Poor glycemic control may contribute to both lower vitamin D status and higher RE susceptibility through inflammation, autonomic dysfunction, altered gastrointestinal motility, and impaired mucosal repair. Together with previous evidence linking vitamin D to insulin resistance, inflammatory regulation, and oxidative stress-related metabolic changes in T2DM, this attenuation is more appropriately interpreted as overlapping metabolic and inflammatory information rather than as evidence excluding a possible biological role of vitamin D in reflux-related mucosal vulnerability (30, 31). Because of the cross-sectional design, the observed associations of higher HbA1c and lower 25(OH)D with RE do not establish temporal sequence or causality. Accordingly, HbA1c and 25(OH)D are interpreted as metabolic correlates of RE rather than causal determinants in this analysis.

### 4.3 Insulin-related metabolic phenotype

HbA1c remains the most stable metabolic correlate of RE across the main models, substitution analyses, and exploratory nonlinear analyses. This finding is clinically relevant, but it should not be interpreted as reflecting a single direct glycemic pathway. Chronic hyperglycemic exposure may contribute to reflux-related mucosal injury through several overlapping mechanisms. First, poor glycemic control may coexist with diabetic autonomic neuropathy and other neurofunctional abnormalities, including altered esophageal motility, lower esophageal sphincter dysfunction, delayed gastric emptying, and impaired esophageal clearance. Previous physiological studies using esophageal manometry, impedance-pH monitoring, and gastric motility assessment in patients with diabetes have suggested that esophageal dysmotility, abnormal reflux burden, delayed clearance, and gastroparesis-related reflux exposure may occur even when reflux symptoms are atypical or incompletely expressed. Second, chronic hyperglycemia may promote oxidative stress, low-grade inflammation, microvascular dysfunction, and impaired epithelial repair, which may increase mucosal vulnerability once reflux exposure occurs. Oxidative damage and inflammatory responses have been implicated in GERD-related epithelial injury and downstream esophageal complications, providing a plausible mechanism through which adverse metabolic status may increase susceptibility to reflux-related mucosal damage (31). Third, the positive associations of fasting insulin, HOMA-IR, and fasting-state ISI in the substitution models, together with the nonlinear spline patterns at higher metabolic ranges, suggest that HbA1c may identify a broader adverse diabetic metabolic phenotype rather than isolated hyperglycemia alone. In this cohort, diabetic neuropathy is more frequent among patients with RE, which is consistent with the biological plausibility that diabetes-related neurofunctional abnormalities may contribute to reflux exposure, prolonged mucosal contact with refluxate, or impaired mucosal defense. However, autonomic function, esophageal motility, gastric emptying, and reflux burden are not directly measured in this dataset; therefore, these mechanisms remain explanatory hypotheses rather than directly tested pathways. The development of endoscopically evident erosive injury likely depends on the combined effects of reflux burden, esophageal clearance, mucosal susceptibility, medication exposure, and metabolic status. At the same time, higher HbA1c may also partly reflect unmeasured lifestyle and behavioral factors associated with reflux risk, rather than indicating a purely glycemic mechanism.

HbA1c remains the most stable metabolic correlate of RE across the main models, substitution analyses, and exploratory nonlinear analyses. This finding is clinically relevant, but it should not be interpreted as reflecting a single direct glycemic pathway. Chronic hyperglycemic exposure may contribute to reflux-related mucosal injury through several overlapping mechanisms. First, poor glycemic control may coexist with diabetic autonomic neuropathy and other neurofunctional abnormalities, including altered esophageal motility, lower esophageal sphincter dysfunction, delayed gastric emptying, and impaired esophageal clearance. This interpretation is supported by evidence that diabetic autonomic neuropathy can affect gastrointestinal function, including delayed esophageal transit and gastroparesis (29). Previous physiological studies using esophageal manometry, impedance-pH monitoring, and gastric motility assessment in patients with diabetes have suggested that esophageal dysmotility, abnormal reflux burden, delayed clearance, and gastroparesis-related reflux exposure may occur even when reflux symptoms are atypical or incompletely expressed (4–6). Second, chronic hyperglycemia may promote oxidative stress, low-grade inflammation, microvascular dysfunction, and impaired epithelial repair, which may increase mucosal vulnerability once reflux exposure occurs. Evidence from T2DM populations also suggests that vitamin D and metabolic status may be related to oxidative stress-related markers, supporting the biological plausibility of a metabolic inflammation-oxidative stress pathway (31). Third, the positive associations of fasting insulin, HOMA-IR, and fasting-state ISI in the substitution models, together with the nonlinear spline patterns at higher metabolic ranges, suggest that HbA1c may identify a broader adverse diabetic metabolic phenotype rather than isolated hyperglycemia alone. In this cohort, diabetic neuropathy is more frequent among patients with RE, which is consistent with the biological plausibility that diabetes-related neurofunctional abnormalities may contribute to reflux exposure, prolonged mucosal contact with refluxate, or impaired mucosal defense. However, autonomic function, esophageal motility, gastric emptying, reflux burden, oxidative stress markers, inflammatory biomarkers, and mucosal repair capacity are not directly measured in this dataset; therefore, these mechanisms remain explanatory hypotheses rather than directly tested pathways. The development of endoscopically evident erosive injury likely depends on the combined effects of reflux burden, esophageal clearance, mucosal susceptibility, medication exposure, and metabolic status. At the same time, higher HbA1c may also partly reflect unmeasured lifestyle and behavioral factors associated with reflux risk, rather than indicating a purely glycemic mechanism.

Insulin-related indicators also show meaningful signals, although these associations should not be interpreted as straightforwardly convergent mechanistic evidence. Fasting insulin, HOMA-IR, and fasting-state ISI are positively associated with RE when entered separately, and the corresponding spline curves suggest stronger associations in higher value ranges. HOMA-IR and fasting insulin primarily reflect insulin resistance and compensatory hyperinsulinemia, whereas fasting-state ISI represents a fasting insulin-to-glucose ratio rather than a dynamic post-load secretion index. Previous studies have linked GERD or erosive esophagitis with metabolic syndrome, insulin resistance, and the TyG index, supporting the interpretation that these insulin-related indicators may reflect a shared adverse metabolic phenotype rather than isolated endocrine abnormalities (10–13, 16, 22, 23). In a cohort with generally poor glycemic control, positive associations across these insulin-related indicators may reflect overlapping features of a dysregulated diabetic metabolic phenotype, rather than a single direct pathway linking insulin resistance or basal insulin secretion to RE. By contrast, the TyG index shows weaker independent contribution after HbA1c adjustment. This may reflect differences among insulin-resistance surrogate markers: TyG captures combined glucose-lipid burden, whereas fasting insulin and HOMA-IR more directly reflect insulin-related metabolic status. The substitution-model strategy helps clarify that the metabolic signal is not driven by a single derived index. Although the sensitivity analyses support the directional consistency of the main metabolic findings, the observed effect sizes are modest and should be interpreted as associations within a hospital-based cross-sectional cohort rather than as evidence of causal or clinically deterministic effects.

Helicobacter pylori status is not independently associated with RE in the adjusted analyses. This null finding should be considered in relation to previous reports suggesting an inverse association between H. pylori infection and erosive esophagitis, particularly in Asian populations where H. pylori prevalence is relatively high. A potential protective association has been attributed to reduced gastric acid secretion in patients with corpus-predominant gastritis or gastric atrophy. In the present cohort, the absence of an independent association may reflect the predominance of diabetes-related metabolic factors, the hospital-based endoscopy setting, prior eradication history, or the lack of detailed information on H. pylori phenotype and gastric atrophy. This result does not exclude a potential protective role of H. pylori in other populations.

### 4.4 Risk stratification and clinical implications

The exploratory discrimination analyses show that adding HbA1c improves model performance, whereas the TyG-based model alone has limited discriminatory value. The augmented model achieves the highest performance, but the overall discrimination remains moderate and calibration is not ideal. These results indicate that routine clinical and laboratory variables contain some risk-stratification information but are insufficient for reliable individual prediction.

Male sex and older age remain positively associated with RE in the main multivariable models. These demographic associations may partly reflect differences in reflux susceptibility, healthcare-seeking behavior, endoscopic referral patterns, or unmeasured lifestyle factors, and should therefore be interpreted cautiously. By contrast, the association for BMI is attenuated after additional clinical and metabolic adjustment, suggesting that the BMI-related signal may overlap with metabolic syndrome, glycemic status, insulin-related abnormalities, and other clinical factors rather than reflecting BMI alone. Clinically, the findings suggest that RE in patients with type 2 diabetes should not be viewed only as an isolated upper gastrointestinal condition. It appears to cluster with poor glycemic control, insulin-related metabolic abnormalities, and lower vitamin D status. These findings may support closer clinical attention to reflux-related mucosal injury in patients with persistently poor glycemic control or adverse metabolic profiles, particularly when reflux symptoms, atypical upper gastrointestinal symptoms, anemia, weight loss, or other clinical indications for endoscopy are present. However, the present study does not establish that elevated HbA1c alone should serve as an indication for routine endoscopic evaluation, nor does it prove that RE is an end-organ complication of chronic hyperglycemia. Rather, endoscopy-confirmed RE may represent one component of the broader clinical burden associated with poorly controlled T2DM. From a clinical management perspective, documented RE in a patient with poor glycemic control may provide an additional opportunity for risk communication, optimization of metabolic status, and collaboration between endocrinology and gastroenterology. Nevertheless, this study does not establish that improving HbA1c, insulin resistance, or vitamin D status reduces RE risk. Prospective studies are needed before these findings can be translated into specific endoscopic screening strategies or intervention recommendations.

### 4.5 Strengths, limitations, and future directions

This study focuses on patients with T2DM and uses endoscopy-based diagnosis to define reflux esophagitis, thereby reducing outcome misclassification caused by symptom-based assessment alone. The analysis integrates clinical characteristics, diabetic complications, glycemic indices, insulin-related indicators, lipid metabolism, renal function, 25(OH)D, and gastrointestinal test results. In addition, prespecified multivariable models, recalculated derived indices, insulin-related indicator substitution, exploratory discrimination analysis, and restricted cubic spline modeling are used to improve analytical consistency and assess the robustness of the findings.

Several limitations remain. Despite consecutive screening, the analytic cohort is derived from a single-center hospital-based endoscopy population of patients with T2DM managed in the Department of Endocrinology from July 1, 2021, to December 31, 2025, rather than a population-based T2DM cohort. Because endoscopy is not routinely performed in all patients with T2DM, selection related to clinical attendance, endoscopic indication, and referral filtering remains possible. The relatively high observed RE prevalence may partly reflect this hospital-based endoscopy setting, because patients with gastrointestinal symptoms, anemia, weight loss, clinical referral indications, or more complex metabolic profiles are more likely to undergo endoscopic evaluation, whereas patients with mild or asymptomatic disease may not be captured. In addition, the relatively high HbA1c levels, particularly among patients with RE, indicate that this cohort may overrepresent hospital-referred or inpatient patients with suboptimally controlled diabetes. These features limit the generalizability of the findings to community-based patients with T2DM or patients with better glycemic control. Information on the specific indication for upper gastrointestinal endoscopy and reflux symptom profile is not consistently available as structured data in the retrospective database. Therefore, this study cannot reliably distinguish patients undergoing endoscopy for typical reflux symptoms, atypical upper gastrointestinal symptoms, dyspepsia, anemia, weight loss, surveillance, or incidental clinical indications. Symptom severity, alarm features, and asymptomatic RE cannot be systematically classified. As a result, the relationship among symptom status, HbA1c, and endoscopy-confirmed RE cannot be directly evaluated, and effect modification by symptomatic versus asymptomatic presentation cannot be assessed. The generalizability of the findings to specific clinical presentations, such as typical symptomatic GERD, atypical reflux symptoms, or incidental/asymptomatic RE, remains uncertain. Exclusion of records without ascertainable RE status or complete prespecified covariate data may introduce bias if missingness is related to metabolic status, endoscopic indication, or RE status. Although conditions such as malignancy, chronic liver disease, connective tissue disease, and pregnancy are considered during data verification when identifiable, residual confounding related to incompletely captured comorbid conditions cannot be excluded. Although the overall number of patients not included in the analytic cohort is reported, criterion-specific exclusion counts could not be reliably reconstructed because the retrospective data extraction did not retain a mutually exclusive stepwise screening log.

The cross-sectional design precludes causal inference and does not establish the temporal relationships among glycemic control, vitamin D status, insulin-related abnormalities, and RE. Reverse causation also remains possible, because RE-related symptoms, dietary modification, or changes in food intake may influence glycemic regulation and metabolic measurements. Unmeasured common causes, including medication exposure, lifestyle factors, nutritional status, and healthcare-seeking behavior, may also contribute to the observed associations. RE is analyzed as a binary outcome because higher-grade LA categories are sparsely distributed, with only one LA-D case; this precludes stable severity-stratified analyses and may obscure grade-specific metabolic associations or nonlinear patterns. Hypertension is modeled as a binary covariate, without further distinction between controlled and uncontrolled hypertension. Residual confounding related to blood pressure control remains possible. Medication exposure is not fully captured and represents an important source of potential outcome misclassification and residual confounding. Proton pump inhibitor use may suppress endoscopic evidence of RE, leading to misclassification of treated RE cases as non-RE. Glucose-lowering therapies, including insulin therapy, glucagon-like peptide-1 receptor agonists, sodium-glucose cotransporter-2 inhibitors, and metformin, may influence HbA1c, insulin-related parameters, body weight, or gastrointestinal motility and thereby affect the observed metabolic associations. Information on vitamin D supplementation is also unavailable and may influence measured 25(OH)D levels, limiting interpretation of the association between vitamin D status and RE. Lifestyle factors, including smoking, alcohol intake, dietary habits, physical activity, sleep posture, and sunlight exposure, are unavailable. Season of 25(OH)D measurement is also not adjusted for, and residual confounding remains possible in the interpretation of the HbA1c-RE and 25(OH)D-RE associations.

Finally, the exploratory model-performance analyses show only modest discrimination and imperfect calibration, and rely on internal validation only. Clinical utility remains uncertain and requires external validation, recalibration, and assessment of decision-analytic benefit. Future prospective, multicenter studies with standardized endoscopic grading, standardized symptom assessment, explicit recording of endoscopic indications, more complete medication and lifestyle exposure assessment, and longitudinal metabolic measurements are needed to clarify temporal relationships and clinical implications.

## 5 Conclusion

This real-world cross-sectional study examines the clinical-metabolic correlates of reflux esophagitis in patients with T2DM using endoscopy-based RE classification and routinely available clinical data. RE is associated with a less favorable metabolic profile, including poorer glycemic control, higher insulin-related metabolic burden, higher triglyceride levels, and lower 25(OH)D levels. Among these factors, HbA1c shows the most consistent association with RE. The inverse association between 25(OH)D and RE is observed before full adjustment for HbA1c but becomes weaker after glycemic control is considered, suggesting that vitamin D status should be interpreted cautiously as a metabolic or behavioral correlate rather than as an independent protective factor.

The present findings support an associational interpretation of the clinical-metabolic profile of RE in patients with T2DM. Higher HbA1c is the most consistently observed metabolic correlate, whereas lower 25(OH)D may reflect metabolic or behavioral differences rather than an independent protective association. Because of the cross-sectional design, reverse causation and unmeasured common causes cannot be excluded; RE-related symptoms or dietary changes may also influence eating behavior and glucose regulation. These findings are hypothesis-generating, and prospective studies incorporating medication exposure, lifestyle factors, vitamin D supplementation, standardized endoscopic grading, and longitudinal metabolic assessment are needed to clarify temporal relationships and clinical implications.

## Data Availability

The raw data supporting the conclusions of this article will be made available by the authors, without undue reservation.
