# Supplementary material for: Metabolic correlates of endoscopy-confirmed reflux esophagitis in hospital-based patients with type 2 diabetes mellitus
Source: Front Endocrinol (Lausanne). 2026 Aug 3;17:1886576. doi: 10.3389/fendo.2026.1886576 (PMC13477878; doi:10.3389/fendo.2026.1886576)
Supplement: Supplementary file 1 [file Supplementaryfile1.docx]

**Supplementary Materials**

(1) Supplementary Figures

Absolute standardized mean differences are presented to describe baseline differences between patients with and without reflux esophagitis.

Figure S1. Standardized mean differences of baseline variables according to reflux esophagitis status.

Spearman correlation coefficients are shown for major continuous metabolic variables. This analysis supports the use of alternative insulin-related models rather than simultaneous inclusion of highly correlated indicators in the same model.

Figure S2. Spearman correlation matrix of major continuous metabolic variables.

Adjusted odds ratios and 95% confidence intervals are shown for all covariates included in Model 5. Continuous variables are standardized and reported per 1-SD increase; binary variables are modeled as yes versus no.

(2) Supplementary Tables

Continuous variables are standardized and reported per 1-SD increase. Binary variables are modeled as yes versus no. False discovery rate q values are calculated using the Benjamini-Hochberg method.

Table S1. Exploratory univariate logistic regression analyses for reflux esophagitis.

| **Predictor** | **OR (95% CI)** | **P value** | **FDR q value** |
| --- | --- | --- | --- |
| HbA1c | 1.55 (1.33-1.81) | <0.001 | <0.001 |
| Fasting insulin | 1.28 (1.10-1.49) | 0.002 | 0.025 |
| Insulin secretion index | 1.26 (1.09-1.47) | 0.002 | 0.025 |
| HOMA-IR | 1.27 (1.08-1.48) | 0.003 | 0.027 |
| Metabolic syndrome | 1.53 (1.12-2.10) | 0.008 | 0.051 |
| 25(OH)D | 0.81 (0.69-0.96) | 0.014 | 0.077 |
| Male sex | 1.44 (1.04-1.99) | 0.029 | 0.107 |
| TyG index | 1.19 (1.02-1.39) | 0.030 | 0.107 |
| BMI | 1.18 (1.01-1.37) | 0.033 | 0.107 |
| Diabetic neuropathy | 1.43 (1.02-2.01) | 0.038 | 0.107 |
| Hypertension | 1.41 (1.02-1.95) | 0.038 | 0.107 |
| Age | 1.18 (1.01-1.39) | 0.040 | 0.107 |
| Triglycerides | 1.15 (1.00-1.34) | 0.055 | 0.134 |
| T3 | 1.14 (0.98-1.33) | 0.093 | 0.213 |
| T4 | 0.90 (0.77-1.06) | 0.213 | 0.454 |
| Fasting plasma glucose | 1.01 (0.86-1.18) | 0.909 | 0.961 |
| eGFR | 0.97 (0.83-1.13) | 0.686 | 0.961 |
| LDL-C | 0.92 (0.79-1.08) | 0.326 | 0.652 |
| Uric acid | 0.94 (0.80-1.10) | 0.424 | 0.752 |
| Fecal occult blood positive | 1.19 (0.78-1.81) | 0.414 | 0.752 |
| Total cholesterol | 0.94 (0.80-1.10) | 0.446 | 0.752 |
| Creatinine | 1.02 (0.87-1.19) | 0.789 | 0.961 |
| BUN | 0.95 (0.81-1.11) | 0.507 | 0.812 |
| HDL-C | 1.05 (0.89-1.22) | 0.578 | 0.881 |
| Homocysteine | 1.03 (0.88-1.20) | 0.725 | 0.961 |
| TSH | 1.02 (0.87-1.19) | 0.788 | 0.961 |
| Fasting C-peptide | 1.00 (0.85-1.16) | 0.961 | 0.961 |
| Diabetes duration | 0.98 (0.84-1.15) | 0.811 | 0.961 |
| Diabetic nephropathy | 0.97 (0.70-1.33) | 0.834 | 0.961 |
| HP positive | 1.04 (0.73-1.48) | 0.842 | 0.961 |
| Diabetic retinopathy | 1.02 (0.66-1.56) | 0.941 | 0.961 |
| Diabetic vascular complications | 1.01 (0.67-1.54) | 0.955 | 0.961 |

Odds ratios are estimated using clinically interpretable increments for selected continuous variables, including HbA1c, 25(OH)D, age, BMI, triglycerides, fasting insulin, insulin secretion index, eGFR, homocysteine, and fasting C-peptide.

Table S2. Clinically interpretable univariate effect estimates for selected continuous predictors.

| **Predictor (increment)** | **OR (95% CI)** | **P value** |
| --- | --- | --- |
| HbA1c (per 1 %) | 1.28 (1.20-1.36) | <0.001 |
| TyG index (per 1 unit) | 1.30 (1.05-1.62) | 0.018 |
| HOMA-IR (per 1 unit) | 1.03 (1.00-1.06) | 0.033 |
| BMI (per 1 kg/m²) | 1.04 (1.00-1.09) | 0.033 |
| 25(OH)D (per 5 ng/mL) | 0.88 (0.78-1.00) | 0.048 |
| Age (per 10 years) | 1.16 (1.01-1.34) | 0.040 |
| Triglycerides (per 1 mmol/L) | 1.08 (1.00-1.16) | 0.052 |
| Fasting insulin (per 10 µIU/mL) | 1.10 (1.00-1.22) | 0.060 |
| ISI (per 1 unit) | 1.06 (0.99-1.14) | 0.083 |
| Fasting plasma glucose (per 1 mmol/L) | 1.04 (0.97-1.11) | 0.249 |
| eGFR (per 10 mL/min/1.73 m²) | 0.98 (0.94-1.02) | 0.275 |
| Homocysteine (per 5 µmol/L) | 1.02 (0.90-1.16) | 0.754 |
| Fasting C-peptide (per 1 ng/mL) | 1.03 (0.83-1.28) | 0.793 |

| **Predictor (increment)** | **OR (95% CI)** | **P value** |
| --- | --- | --- |
| HbA1c (per 1%) | 1.17 (1.11-1.24) | <0.001 |
| TyG index (per 1 unit) | 1.29 (1.02-1.61) | 0.030 |
| HOMA-IR (per 1 unit) | 1.07 (1.02-1.11) | 0.003 |
| BMI (per 1 kg/m²) | 1.04 (1.00-1.09) | 0.033 |
| 25(OH)D (per 5 ng/mL) | 0.86 (0.76-0.97) | 0.014 |
| Age (per 10 years) | 1.16 (1.01-1.34) | 0.040 |
| Triglycerides (per 1 mmol/L) | 1.08 (1.00-1.16) | 0.055 |
| Fasting insulin (per 10 μIU/mL) | 1.26 (1.09-1.46) | 0.002 |
| Insulin secretion index (per 1 unit) | 1.17 (1.06-1.29) | 0.002 |
| Fasting plasma glucose (per 1 mmol/L) | 1.01 (0.90-1.13) | 0.909 |
| eGFR (per 10 mL/min/1.73 m²) | 0.99 (0.93-1.05) | 0.686 |
| Homocysteine (per 5 μmol/L) | 1.03 (0.88-1.21) | 0.725 |
| Fasting C-peptide (per 1 ng/mL) | 0.99 (0.79-1.25) | 0.961 |

Full estimates are provided for all covariates included in Models 1-5. Continuous variables are standardized and reported per 1-SD increase; binary variables are modeled as yes versus no.

Table S3. Full prespecified multivariable logistic regression models for reflux esophagitis.

| **Predictor** | **Model 1 aOR (95% CI)** | **P** | **Model 2 aOR (95% CI)** | **P** | **Model 3 aOR (95% CI)** | **P** | **Model 4 aOR (95% CI)** | **P** | **Model 5 aOR (95% CI)** | **P** |
| --- | --- | --- | --- | --- | --- | --- | --- | --- | --- | --- |
| Male sex | 1.45 (1.04-2.01) | 0.026 | 1.44 (1.01-2.06) | 0.044 | 1.53 (1.07-2.19) | 0.021 | 1.52 (1.05-2.19) | 0.026 | 1.51 (1.04-2.19) | 0.029 |
| Age | 1.24 (1.05-1.46) | 0.012 | 1.22 (1.01-1.48) | 0.039 | 1.22 (1.01-1.48) | 0.037 | 1.24 (1.03-1.50) | 0.027 | 1.24 (1.03-1.50) | 0.026 |
| Diabetes duration | 0.96 (0.82-1.14) | 0.659 | 0.95 (0.78-1.15) | 0.600 | 0.94 (0.77-1.14) | 0.514 | 0.98 (0.80-1.20) | 0.843 | 0.98 (0.80-1.20) | 0.841 |
| BMI | 1.21 (1.03-1.43) | 0.020 | 1.16 (0.98-1.37) | 0.082 | 1.15 (0.97-1.36) | 0.111 | 1.12 (0.94-1.33) | 0.193 | 1.12 (0.94-1.33) | 0.204 |
| Hypertension | - | - | 1.29 (0.90-1.84) | 0.161 | 1.21 (0.84-1.73) | 0.298 | 1.34 (0.93-1.95) | 0.121 | 1.35 (0.93-1.96) | 0.119 |
| Diabetic nephropathy | - | - | 0.86 (0.60-1.23) | 0.407 | 0.91 (0.63-1.31) | 0.611 | 0.86 (0.59-1.25) | 0.425 | 0.86 (0.59-1.24) | 0.421 |
| Diabetic retinopathy | - | - | 0.92 (0.57-1.47) | 0.720 | 0.91 (0.57-1.46) | 0.700 | 0.74 (0.45-1.21) | 0.231 | 0.74 (0.45-1.21) | 0.231 |
| Diabetic neuropathy | - | - | 1.34 (0.91-1.96) | 0.138 | 1.36 (0.93-2.00) | 0.116 | 1.25 (0.84-1.86) | 0.268 | 1.25 (0.84-1.86) | 0.264 |
| Diabetic vascular complications | - | - | 0.81 (0.50-1.30) | 0.383 | 0.79 (0.49-1.28) | 0.343 | 0.83 (0.51-1.36) | 0.467 | 0.83 (0.51-1.36) | 0.466 |
| eGFR | - | - | 0.99 (0.83-1.18) | 0.911 | 0.99 (0.83-1.18) | 0.933 | 0.97 (0.81-1.16) | 0.736 | 0.97 (0.81-1.16) | 0.735 |
| Fecal occult blood positive | - | - | 0.99 (0.63-1.54) | 0.963 | 0.98 (0.63-1.52) | 0.916 | 1.01 (0.64-1.60) | 0.953 | 1.01 (0.64-1.60) | 0.955 |
| HP positive | - | - | 1.13 (0.78-1.64) | 0.513 | 1.16 (0.80-1.69) | 0.425 | 1.25 (0.86-1.84) | 0.246 | 1.25 (0.86-1.84) | 0.246 |
| 25(OH)D | - | - | - | - | 0.80 (0.68-0.96) | 0.013 | 0.84 (0.70-1.00) | 0.051 | 0.84 (0.70-1.00) | 0.052 |
| HbA1c | - | - | - | - | - | - | 1.58 (1.34-1.86) | <0.001 | 1.58 (1.33-1.87) | <0.001 |
| TyG index | - | - | - | - | - | - | - | - | 1.01 (0.85-1.21) | 0.869 |

The TyG-based model and the additional HbA1c-adjusted model are presented as supplementary analyses to facilitate comparison with the earlier modeling framework.

Table S4. Alternative multivariable models using the TyG index as the insulin-resistance surrogate.

| **Predictor** | **TyG-based model aOR (95% CI)** | **P value** | **Additional HbA1c-adjusted model aOR (95% CI)** | **P value** |
| --- | --- | --- | --- | --- |
| Male sex | 1.38 (0.98-1.95) | 0.067 | 1.39 (0.98-1.98) | 0.065 |
| Age | 1.18 (1.00-1.41) | 0.053 | 1.20 (1.01-1.43) | 0.034 |
| Diabetes duration | 0.90 (0.75-1.08) | 0.253 | 0.92 (0.77-1.10) | 0.374 |
| BMI | 1.13 (0.93-1.37) | 0.215 | 1.13 (0.94-1.37) | 0.198 |
| Hypertension | 1.21 (0.81-1.80) | 0.354 | 1.27 (0.85-1.92) | 0.246 |
| Diabetic neuropathy | 1.31 (0.90-1.92) | 0.157 | 1.21 (0.82-1.78) | 0.348 |
| Metabolic syndrome | 1.03 (0.64-1.65) | 0.915 | 1.01 (0.62-1.65) | 0.961 |
| TyG index | 1.14 (0.94-1.39) | 0.195 | 1.00 (0.82-1.23) | 0.963 |
| 25(OH)D | 0.81 (0.68-0.96) | 0.017 | 0.84 (0.70-0.99) | 0.043 |
| HbA1c | - | - | 1.54 (1.30-1.82) | <0.001 |

Variance inflation factors are calculated to assess multicollinearity among major covariates included in the multivariable and sensitivity models.

Table S5. Variance inflation factors for major covariates.

| **Variable** | **TyG model** | **HOMA-IR model** | **Fasting insulin model** | **Insulin secretion index model** |
| --- | --- | --- | --- | --- |
| Male sex | 1.22 | 1.21 | 1.21 | 1.21 |
| Age | 1.45 | 1.45 | 1.45 | 1.46 |
| Diabetes duration | 1.53 | 1.53 | 1.53 | 1.53 |
| BMI | 1.20 | 1.22 | 1.24 | 1.24 |
| Hypertension | 1.22 | 1.24 | 1.23 | 1.22 |
| Diabetic nephropathy | 1.29 | 1.29 | 1.29 | 1.29 |
| Diabetic retinopathy | 1.22 | 1.22 | 1.22 | 1.22 |
| Diabetic neuropathy | 1.29 | 1.29 | 1.29 | 1.29 |
| Diabetic vascular complications | 1.27 | 1.29 | 1.30 | 1.31 |
| eGFR | 1.23 | 1.23 | 1.22 | 1.23 |
| Fecal occult blood positive | 1.11 | 1.11 | 1.11 | 1.11 |
| HP positive | 1.06 | 1.06 | 1.06 | 1.06 |
| 25(OH)D | 1.08 | 1.09 | 1.09 | 1.08 |
| HbA1c | 1.17 | 1.08 | 1.07 | 1.07 |
| TyG index | 1.18 | - | - | - |
| HOMA-IR | - | 1.14 | - | - |
| Fasting insulin | - | - | 1.17 | - |
| Insulin secretion index | - | - | - | 1.16 |

Table S6. Categorical analysis of vitamin D status and reflux esophagitis

| **Vitamin D status** | **Total n** | **RE n (%)** | **Non-RE n (%)** | **Model VD-1 OR (95% CI), P** | **Model VD-2 OR (95% CI), P** | **Model VD-3 OR (95% CI), P** | **Model VD-4 OR (95% CI), P** |
| --- | --- | --- | --- | --- | --- | --- | --- |
| Sufficient, ≥30 ng/mL | 49 | 9 (18.4) | 40 (81.6) | 1.00 (reference) | 1.00 (reference) | 1.00 (reference) | 1.00 (reference) |
| Insufficient, 20 to <30 ng/mL | 209 | 52 (24.9) | 157 (75.1) | 1.38 (0.63-3.06), P=0.421 | 1.43 (0.64-3.20), P=0.388 | 1.38 (0.61-3.13), P=0.444 | 1.37 (0.60-3.12), P=0.452 |
| Deficient, <20 ng/mL | 514 | 162 (31.5) | 352 (68.5) | 1.97 (0.93-4.17), P=0.078 | 2.01 (0.93-4.33), P=0.075 | 1.77 (0.81-3.88), P=0.153 | 1.76 (0.80-3.86), P=0.156 |
| P for trend | - | - | - | 0.017 | 0.019 | 0.074 | 0.075 |

Values are shown as n (%) or OR (95% CI). Model VD-1 adjusts for age, sex, diabetes duration, and BMI. Model VD-2 further adjusts for hypertension, diabetic nephropathy, diabetic retinopathy, diabetic neuropathy, diabetic vascular complications, eGFR, fecal occult blood, and HP status. Model VD-3 further adjusts for HbA1c, and Model VD-4 further adjusts for the TyG index. The sufficient group is used as the reference category. P for trend is calculated using an ordinal 25(OH)D status variable coded as sufficient=0, insufficient=1, and deficient=2.

Table S7. Variance inflation factors for predictors included in the main multivariable models and sensitivity models

| **Predictor** | **Model 1** | **Model 2** | **Model 3** | **Model 4** | **Model 5** | **Sensitivity model A** | **Sensitivity model B** | **Sensitivity model C** |
| --- | --- | --- | --- | --- | --- | --- | --- | --- |
| Age | 1.08 | 1.44 | 1.44 | 1.45 | 1.45 | 1.45 | 1.45 | 1.46 |
| Sex | 1.01 | 1.18 | 1.20 | 1.20 | 1.22 | 1.21 | 1.21 | 1.21 |
| Diabetes duration | 1.11 | 1.52 | 1.52 | 1.53 | 1.53 | 1.53 | 1.53 | 1.53 |
| Body mass index | 1.06 | 1.16 | 1.16 | 1.17 | 1.20 | 1.22 | 1.24 | 1.24 |
| Hypertension | - | 1.18 | 1.20 | 1.21 | 1.22 | 1.24 | 1.23 | 1.22 |
| Diabetic nephropathy | - | 1.26 | 1.29 | 1.29 | 1.29 | 1.29 | 1.29 | 1.29 |
| Diabetic retinopathy | - | 1.19 | 1.19 | 1.22 | 1.22 | 1.22 | 1.22 | 1.22 |
| Diabetic neuropathy | - | 1.27 | 1.28 | 1.29 | 1.29 | 1.29 | 1.29 | 1.29 |
| Diabetic vascular complications | - | 1.27 | 1.27 | 1.27 | 1.27 | 1.29 | 1.30 | 1.31 |
| Estimated glomerular filtration rate | - | 1.22 | 1.22 | 1.22 | 1.23 | 1.23 | 1.22 | 1.23 |
| Fecal occult blood | - | 1.11 | 1.11 | 1.11 | 1.11 | 1.11 | 1.11 | 1.11 |
| *Helicobacter pylori* status | - | 1.06 | 1.06 | 1.06 | 1.06 | 1.06 | 1.06 | 1.06 |
| 25-hydroxyvitamin D | - | - | 1.07 | 1.08 | 1.08 | 1.09 | 1.09 | 1.08 |
| Glycated hemoglobin | - | - | - | 1.07 | 1.17 | 1.08 | 1.07 | 1.07 |
| Triglyceride-glucose index | - | - | - | - | 1.18 | - | - | - |
| HOMA-IR | - | - | - | - | - | 1.14 | - | - |
| Fasting insulin | - | - | - | - | - | - | 1.17 | - |
| Fasting-state insulin secretion index | - | - | - | - | - | - | - | 1.16 |
